# Clinical practice recommendations for growth hormone treatment in children with chronic kidney disease

**DOI:** 10.1038/s41581-019-0161-4

**Published:** 2019-06-13

**Authors:** Jens Drube, Mandy Wan, Marjolein Bonthuis, Elke Wühl, Justine Bacchetta, Fernando Santos, Ryszard Grenda, Alberto Edefonti, Jerome Harambat, Rukshana Shroff, Burkhard Tönshoff, Dieter Haffner, on behalf of the European Society for Paediatric Nephrology Chronic Kidney Disease Mineral and Bone Disorders, Dialysis, and Transplantation Working Groups

**Affiliations:** 1 Department of Pediatric Kidney, Liver and Metabolic Diseases, Hannover Medical School Children’s Hospital, Hannover, Germany; 2 Center for Congenital Kidney Diseases, Center for Rare Diseases, Hannover Medical School, Hannover, Germany; 3 Renal Unit, Great Ormond Street Hospital for Children, London, UK; 4 ESPN/ERA-EDTA Registry, Department of Medical Informatics, Academic Medical Center, University of Amsterdam, Amsterdam, Netherlands; 5 Department of Pediatrics I, University Children’s Hospital, Heidelberg, Germany; 6 Department of Pediatric Nephrology, Rheumatology and Dermatology, University Children’s Hospital, Lyon, France; 7 Department of Pediatrics, Hospital Universitario Central de Asturias (HUCA), Health Service of the Principality of Asturias, SESPA, Oviedo, Spain; 8 Department of Nephrology, Kidney Transplantation and Arterial Hypertension, The Children’s Memorial Health Institute, Warsaw, Poland; 9 Fondazione IRCCS Ca’ Granda Ospedale Maggiore Policlinico, Milan, Italy; 10 Department of Pediatrics, Pediatric Nephrology Unit, Bordeaux University Hospital, Bordeaux, France

**Keywords:** Growth disorders, Chronic kidney disease, Paediatric kidney disease, Hormones

## Abstract

Achieving normal growth is one of the most challenging problems in the management of children with chronic kidney disease (CKD). Treatment with recombinant human growth hormone (GH) promotes longitudinal growth and likely enables children with CKD and short stature to reach normal adult height. Here, members of the European Society for Paediatric Nephrology (ESPN) CKD–Mineral and Bone Disorder (MBD), Dialysis and Transplantation working groups present clinical practice recommendations for the use of GH in children with CKD on dialysis and after renal transplantation. These recommendations have been developed with input from an external advisory group of paediatric endocrinologists, paediatric nephrologists and patient representatives. We recommend that children with stage 3–5 CKD or on dialysis should be candidates for GH therapy if they have persistent growth failure, defined as a height below the third percentile for age and sex and a height velocity below the twenty-fifth percentile, once other potentially treatable risk factors for growth failure have been adequately addressed and provided the child has growth potential. In children who have received a kidney transplant and fulfil the above growth criteria, we recommend initiation of GH therapy 1 year after transplantation if spontaneous catch-up growth does not occur and steroid-free immunosuppression is not a feasible option. GH should be given at dosages of 0.045–0.05 mg/kg per day by daily subcutaneous injections until the patient has reached their final height or until renal transplantation. In addition to providing treatment recommendations, a cost-effectiveness analysis is provided that might help guide decision-making.

## Abstract (web summary)

This Evidence-Based Guideline developed by members of the European Society for Paediatric Nephrology CKD-MBD, Dialysis and Transplantation working groups presents clinical practice recommendations for the use of growth hormone in children with chronic kidney disease on dialysis and after renal transplantation.

## Introduction

**Fig. 1:**
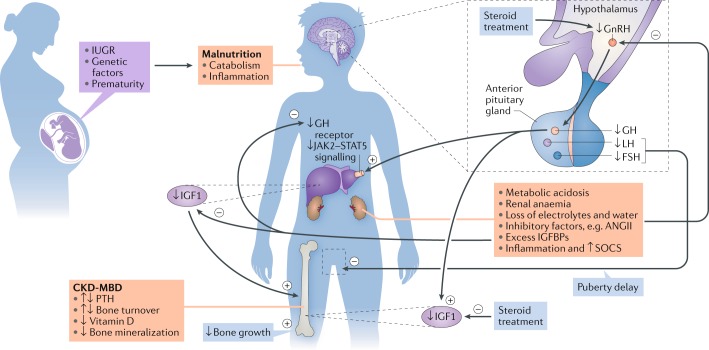
Factors that contribute to growth failure in children with CKD. The aetiology of growth failure in chronic kidney disease (CKD) is multifactorial and includes intrauterine growth restriction (IUGR), genetic factors such as parental height and primary renal disease, prematurity and malnutrition, which especially limits growth in children with congenital CKD. CKD–mineral and bone disorder (CKD-MBD), metabolic acidosis, anaemia, loss of electrolytes and water and disturbances of the somatotropic and gonadotropic hormone axes also contribute to growth failure. CKD is a state of growth hormone (GH) insensitivity that is characterized by deficiency of functional insulin-like growth factor 1 (IGF1) due to reduced GH receptor expression in target organs such as the liver and disturbed GH receptor signalling via the Janus kinase 2 (JAK2)–signal transducer and activator of transcription 5 (STAT5) pathway due to inflammation-induced suppressor of cytokine signalling (SOCS) expression and increased IGF binding capacity due to excess of IGF binding proteins (IGFBPs). Finally, reduced release of hypothalamic gonadotropin-releasing hormone (GnRH), due to uraemia-related inhibitory factors such as angiotensin II (ANGII) and steroid treatment, might result in decreased circulating levels of bioactive luteinizing hormone (LH), hypogonadism and reduced pubertal growth spurt. The GH insensitivity in CKD can be overcome by the administration of supraphysiological doses of recombinant human GH, which stimulates IGF1 synthesis, normalizes somatomedin bioactivity, promotes longitudinal growth and likely improves adult height. FSH, follicle-stimulating hormone; PTH, parathyroid hormone.

In the past two decades, major progress has been made in our understanding of the pathophysiology and treatment of growth failure in children with chronic kidney disease (CKD). Nevertheless, approximately 40% of children with end-stage renal disease (ESRD) have a reduced final height (below the third percentile) compared with that of healthy age-matched and sex-matched controls^1,2^. Short stature impairs quality of life, self-esteem and social rehabilitation and is associated with increased mortality^3–6^. The aetiology of growth failure in CKD is multifactorial and includes intrauterine growth restriction, malnutrition, mineral and bone disorder (MBD), metabolic acidosis, loss of electrolytes and disturbances of the somatotropic and gonadotropic hormone axes (Fig. 1). In particular, advanced CKD is a state of growth hormone (GH) insensitivity, characterized by deficiency of functional insulin-like growth factor 1 (IGF1)^7^. This GH insensitivity can be overcome by the administration of supraphysiological doses of recombinant human GH (abbreviated to GH hereafter), which stimulates IGF1 synthesis, normalizes somatomedin bioactivity, promotes longitudinal growth and likely improves adult height^8,9^. Consequently, GH has been licensed for the treatment of CKD-induced growth failure in Europe, North America and many other high-income countries.

However, other than a treatment algorithm proposed by members of a consensus conference held in 2003 (ref.^10^) and a brief guidance from the Kidney Disease Outcomes Quality Initiative (KDOQI)^11^, no comprehensive evidence-based recommendations are available for the use of GH in children with CKD. To address this gap, members of the European Society for Paediatric Nephrology (ESPN) CKD-MBD, Dialysis and Transplantation working groups have developed clinical practice recommendations (CPRs) for the use of GH in children with CKD on dialysis and after renal transplantation with input from experts in paediatric nephrology, paediatric endocrinology, pharmacy, epidemiology and patient representatives. Recommendations are based on evidence where possible. In the absence of suitable evidence, the opinion of experts from the working groups is provided but clearly graded as such and must be carefully considered by the treating physician and adapted to individual patient needs as appropriate. Although the CPRs have been generated by a European society, they are intended to be useful to clinicians within and beyond Europe. These CPRs will be audited and revised periodically. Research recommendations to study key GH outcome measures in children with CKD are also suggested.

## Methods

### Overview of the guideline project

We have followed the RIGHT (Reporting Items for practice Guidelines in Healthcare) Statement for Practice Guidelines^12^. To generate this Evidence-Based Guideline, three groups were assembled: a core leadership group, an external advisory panel and a voting panel. The core group comprised paediatric nephrologists who are members of the ESPN CKD-MBD, Dialysis and Transplantation working groups, a paediatric pharmacist and an epidemiologist. The external advisory group included two paediatric endocrinologists, four patient representatives and two additional paediatric nephrologists with expertise in GH treatment in children. The patient representatives discussed the manuscript provided by the core group members within their local parents’ association and their suggestions were incorporated into the manuscript by members of the core leadership group. The voting group included 37 members of the ESPN CKD-MBD, Dialysis and Transplantation working groups with expertise in paediatric CKD. Voting group members were asked by use of an e-questionnaire to provide a level of agreement on a five-point scale (strongly disagree, disagree, neither agree/disagree, agree and strongly agree) (Delphi method). Failing a 70% level of consensus, recommendations were modified after discussion by the core group and reviewed again by the voting panel until a consensus level of at least 70% was achieved.

### Developing the PICO questions

We developed PICO (Patient or Population covered, Intervention, Comparator, Outcome) questions as follows^13^. These PICO elements were arranged into the questions to be addressed in the literature searches. Each PICO question then formed the basis for a recommendation.

The population covered included children with CKD on dialysis and after transplantation. The intervention and comparators considered included treatment with GH compared with no treatment or placebo. We addressed recommendations for the initiation, continuation and discontinuation of GH therapy, its growth-promoting effects (including biochemical and skeletal effects) and its safety. Although some reports suggest that GH may have anabolic effects and improve muscle strength and quality of life, most of these data are based on preclinical or low-grade association studies and were not considered in this document.

### Literature search

The PubMed database was searched for studies published up to 15 May 2018; all systematic reviews of randomized controlled trials (RCTs) on GH for the promotion of growth in children with CKD for growth and all RCTs, prospective uncontrolled trials, observational studies and registry studies on GH in children with CKD, irrespective of the number of patients, were included. Registry data were used only for final height analyses and safety measures. The following key words were used to identify suitable studies: “chronic renal failure” OR “CKD” OR “chronic renal insufficiency” OR “dialysis” OR “renal transplantation” OR “kidney transplantation”) AND (“children” OR “paediatric” OR “pediatric” OR “child”) AND (“growth hormone” OR “GH”) AND (“treatment” OR “therapy”). Included studies were limited to those published in English, with at least 6 months of follow-up, and containing data on standardized height before and at the end of study, data on CKD stage (as assessed by glomerular filtration rate (GFR) or degree of renal impairment), mode of renal replacement therapy, child participants only (aged 0–18 years) and with at least five GH-treated patients. The search retrieved 530 results, and 62 articles are referenced here.

Further details and a summary of the publications used for this CPR are given in the Supplementary material (Supplementary Tables 1–4).

### Grading system

**Fig. 2:**
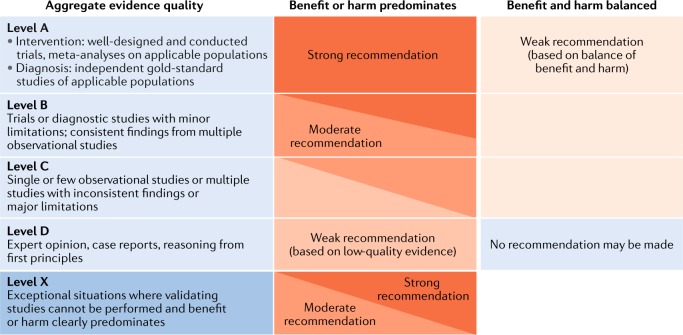
Matrix for the grading of evidence and assigning strength of recommendations as currently used by the American Academy of Pediatrics. Reproduced with permission from ref.^14^: *Pediatrics* **140**, e20171904 Copyright © 2017 by the AAP.

We have followed the grading system of the American Academy of Pediatrics to develop the recommendations^14^ (Fig. 2). The quality of evidence is graded high (A), moderate (B), low (C), very low (D) or not applicable (X). The latter grading (X) refers to exceptional situations in which validating studies cannot be performed and benefit or harm clearly predominates and was used to grade contraindications for GH use and safety parameters. The strength of a recommendation is graded strong, moderate, weak or discretionary (when no recommendation can be made).

### Cost-effectiveness analysis

A cost-effectiveness analysis was performed on the basis of an expected gain in final height of 7.2 cm given treatment for 2–5 years and an average cost of €22 per 1 mg GH in two clinical scenarios: a child aged 5 years at the start of GH therapy and an adolescent aged 12 years at the start of GH therapy, both in the twenty-fifth percentile for age-related and sex-related weight (Supplementary Box 1; Supplementary Tables 5, 6).

### Limitations of the guideline process

Most RCTs of GH use in children with CKD were performed in the 1990s when the conduct of trials was not as robust as that of current trials. For example, many of these RCTs did not include all enrolled participants in their analyses. In addition, the size of many RCTs was small owing to the low incidence of childhood CKD; thus, the strength of most recommendations is weak to moderate. Owing to the limited budget of this ESPN initiative, the core group did not include physicians outside Europe or patient representatives. The lack of patient representatives in the core group was partly mitigated by including patient representatives from Germany, Italy, Belgium and the UK as external experts.

## Initial work-up

### Assessment of statural growth

As for any child suffering from a potentially growth-limiting chronic disease, length (before 2 years of age) and height (from 2 years of age) should be assessed regularly by trained personnel using calibrated equipment and standardized techniques and compared with sex-specific and age-specific reference charts for healthy children (Box 1). Gestational age is taken into account when assessing length of infants born prematurely. Supine length is measured using a validated length board or mat up to a length of 80 cm or if assessment of standing height is not feasible. In older children, standing height is measured using a wall-mounted stadiometer^11^.

The frequency of monitoring is based on the patient’s age and stage of CKD (Table 1). Children with evidence of growth delay, comorbidities including central nervous system, liver or heart involvement and rapid changes in health status or dietary intake might warrant more frequent assessment than those with milder or more stable disease^11,15,16^. The calculation of height velocity over a minimum follow-up of 6 months is a more sensitive index of growth than a single height measurement^17^. This approach enables the identification of children with reduced growth rates (that is, those below the twenty-fifth percentile) who might benefit from growth-promoting measures. By contrast, a growth velocity above the seventy-fifth percentile indicates catch-up growth^18^. Optimal growth monitoring requires updated national growth charts. If unavailable, northern or southern European growth charts are preferable over other national or international growth charts for European children with CKD^19^.

**Table 1:** Assessment intervals for statural growth in CKD

Assessment type	Age (years)	Recommended intervals of assessment (months)			
		CKD stage 3	CKD stage 4	CKD stage 4–5	CKD stage 5D
Length^a^ or height	0–1	0.5–2	0.5–2	0.5–2	0.5–2
	1–3	1–3	1–2	1–2	1–2
	>3	3–6	1–3	1–3	1–3
Length velocity^a^ or height velocity	0–1	0.5–2	0.5–2	0.5–2	0.5–1
	1–3	1–6	1–3	1–3	1–2
	>3	6	6	6	6

Recommendations were generated by combining those from the Kidney Disease Outcomes Quality Initiative (KDOQI) guidelines; the Caring for Australasians with Renal Impairment (CARI) guidelines; and the Clinical Guideline from the British Society for Paediatric Endocrinology and Diabetes (BSPED), the British Association for Paediatric Nephrology (BAPN) and the Paediatric Renal Interest Nutrition Group (PRING)^11^^,^^15^^,^^16^. CKD, chronic kidney disease. ^a^Supine length is measured using a validated length board or mat up to a length of 80 cm (before 2 years of age) or if assessment of standing height is not feasible.

### Assessment of growth potential

Assessment of the extent to which the epiphysis of the left wrist is open on a radiography image is the standard approach for assessing a child’s growth potential^20^. The calculation of genetic target height (T_H_, in cm) can be performed using Tanner’s formula for girls (equation 1) and for boys (equation 2):

1 $$\frac{{H}_{{\rm{mother}}}+{H}_{{\rm{father}}}-13}{2}$$

2 $$\frac{{H}_{{\rm{mother}}}+{H}_{{\rm{father}}}+13}{2}$$

These equations are based on mid-parental height and assume that the parents do not have a chronic disease^21^. Alternatively, the Molinari formula can be used for girls (equation 3) and for boys (equation 4):

3 $$\frac{{H}_{{\rm{mother}}}+{H}_{{\rm{father}}}}{2}-2.6$$

4 $$\frac{{H}_{{\rm{mother}}}+{H}_{{\rm{father}}}}{2}+10.2$$

These equations assume a secular trend across generations of 3.8 cm (ref.^22^). The application of adult height prediction methods such as the Tanner–Whitehouse Mark II method is not generally recommended for children with CKD because several validation studies have shown that these formulae overestimated final height by 3–10 cm (refs^23,24^).

### CKD-associated growth-limiting factors

The pathogenesis of growth retardation in CKD is multifactorial (Box 2). The two most important factors are CKD severity and age at disease onset. In order to maximize the growth-promoting effect of GH and to reduce the risk of adverse events, CKD-associated factors that adversely interfere with growth or affect the therapeutic response need to be adequately addressed before GH treatment is initiated.

#### Nutrition

Nutritional deficiency is a major cause of growth failure and is particularly important in infancy and early childhood, which represent the most nutrition-sensitive phases of growth^25^. Prescription of a nutritionally appropriate feed, if needed via gastrostomy or nasogastric tube, is therefore recommended to optimize growth^26^ before GH treatment is considered^11^. Optimal nutritional management might result in catch-up growth in infants even with severe CKD^27,28^.

#### Hormonal disturbances

The main hormonal disturbance that leads to growth failure in CKD is GH insensitivity (described earlier). Pubertal delay and reduced pubertal growth spurts are characteristic findings of longstanding ESRD and probably result from the reduced release of hypothalamic gonadotropin-releasing hormone (GnRH) and decreased circulating levels of bioactive luteinizing hormone (LH) due to CKD-related inhibitory factors such as angiotensin II^29^. Patients who demonstrate delayed puberty — that is, boys with a testicular volume less than 4 ml at the age of 14 years and girls with breast stage less than B2 at age 13.5 years — should be referred to a paediatric endocrinologist for full work-up and eventual induction of puberty. Hypothyroidism and GH deficiency can cause growth failure in children independent of underlying CKD. Therefore, we recommend that thyroid hormone levels, including serum TSH and free T3, and serum IGF1 levels are measured before GH therapy is initiated. We do not recommend assessment of IGF binding protein 3 (IGFBP3) serum concentrations in patients with CKD because low-molecular-mass immunoreactive IGFBP3 fragments accumulate in CKD^30^ and thus most commercially available IGFBP3 assays deliver falsely elevated IGFBP3 serum levels.

#### Metabolic control

Indirect evidence suggests that good metabolic control is necessary to facilitate longitudinal growth in CKD. Children with isolated tubular disorders resulting in urinary salt, water or bicarbonate losses show severe growth retardation^31–33^. Therefore, we recommend that children with CKD resulting in hyponatraemia and polyuria receive free water and sodium supplementation to avoid chronic intravascular volume depletion and permit optimal growth. Metabolic acidosis should be corrected (to serum bicarbonate levels ≥22 mEq/l) by administration of sodium bicarbonate and/or the use of HCO_3_-based or lactate-based dialysis solutions in children on dialysis^34^.

#### CKD-MBD

Although reduced height velocity is considered a clinical manifestation of CKD-MBD, the relationship of longitudinal growth with serum parathyroid hormone (PTH) and vitamin D values is unclear^35^. Increased serum PTH and vitamin D deficiency might contribute to growth retardation; therefore, marked secondary hyperparathyroidism should be controlled before GH therapy is commenced^9^. Thus, serum PTH levels should be kept within the recommended CKD-stage-dependent target range for children^35–37^. Serum concentrations of total 25-hydroxyvitamin D_3_ should be kept at above 30 ng/ml in all stages of CKD^38^.

#### Adequacy of dialysis

The use of biocompatible peritoneal dialysate is associated with improved height in young children on chronic peritoneal dialysis^39^. Increased protein administration and increased urea clearance were associated with improved growth in well-nourished children receiving haemodialysis even in the absence of GH treatment^40^. Therefore, optimization of dialysis regimens should always be attempted to improve growth.

#### Anaemia

Anaemia may contribute to CKD-related growth failure^41,42^. Target haemoglobin concentrations as recommended by the Kidney Disease Improving Global Outcomes (KDIGO) guidelines should therefore be achieved before starting GH therapy^43^.

#### Comorbidities

Systemic diseases or genetic syndromes that are often associated with paediatric CKD, including Schimke immune-osseous dysplasia, Bardet–Biedl syndrome and Galloway–Mowat syndrome, can interfere markedly with normal growth^27^. These patients have typically been excluded from RCTs of GH therapy; therefore, the efficacy and safety of GH in patients with these conditions have not been evaluated. Coexisting or preceding history of malignancies contraindicate GH therapy.

### Clinical and organ assessments and contraindications

We suggest that all paediatric patients who are being considered for GH therapy undergo a fundoscopic examination to rule out pre-existing papilledema, which could be suggestive of intracranial hypertension that might progress during GH therapy^44^. Radiography of the left wrist is recommended in order to assess bone age to document open epiphyses for the assessment of growth potential, as mentioned above. Assessment of pubertal stage should be performed according to Tanner stages in patients older than 10 years of age. Furthermore, GH must not be started in the case of known hypersensitivity against this drug or its constituent parts, or in patients with an active tumour or uncontrolled diabetes mellitus.

Box 1 Recommendations for work-up before initiation of GH treatment

- We recommend that height (or supine length for patients below 2 years of age) is regularly measured depending on age and chronic kidney disease (CKD) stage (TABLE 1). Height velocity should be calculated over a minimum period of 6 months, and both height and height velocity should be compared with standardized growth charts (grade A, strong recommendation).
- We recommend that growth potential is assessed by calculation of genetic target height on the basis of parental height and the extent to which the epiphysis of the left wrist is open on radiography (grade A, strong recommendation). We do not recommend application of adult height prediction methods for children with CKD (grade C, weak recommendation).
- Age, primary renal disease, systemic disorders, stage of CKD, dialysis adequacy (for patients on dialysis) and graft function and glucocorticoid therapy (in children post-transplantation) should be taken into account when considering growth hormone (GH) therapy (grade B, moderate recommendation).
- CKD-associated growth-limiting factors such as protein-calorie malnutrition, metabolic acidosis, electrolyte disturbances (hyponatraemia), dehydration and mineral dysregulation, including secondary hyperparathyroidism, should be adequately controlled before considering GH therapy (grade A, strong recommendation).
- The following assessments should be performed before starting GH:

- Serum creatinine (and estimated glomerular filtration rate), urea, calcium, phosphorus, total alkaline phosphatase, bicarbonate, parathyroid hormone, 25(OH) vitamin D, albumin, fasting glucose and glycosylated haemoglobin levels
- Serum thyroid hormone (TSH and free T3) and insulin-like growth factor 1 concentrations
- Fundoscopic examination
- Radiography of the left wrist
- Pubertal status according to Tanner (grade C, moderate recommendation)

Box 2 Factors that contribute to growth failure in children with CKD

- Genetic factors

- Parental heights
- Gender
- Syndromic kidney diseases

- Birth-related factors

- Prematurity
- Small for gestational age
- Intensive care requirement

- Comorbidities (for example, central nervous system, liver or heart involvement)
- Age at onset of chronic kidney disease (CKD)
- Severity of CKD and residual renal function in patients on dialysis
- Metabolic disturbances

- Salt and water metabolism
- Metabolic acidosis
- CKD–mineral and bone disorder (MBD)

- Anaemia

- Malnutrition

- Altered taste sensation
- Anorexia
- Vomiting
- Dietary restrictions
- Nutrient losses in dialysate
- Infections and inflammation

- Protein–energy wasting

- Infections and inflammation
- Uraemic toxins
- Oxidative stress
- Inflammatory cytokines

- Hormonal disturbances affecting

- Somatotropic hormone axis
- Gonadotropic hormone axis
- Parathyroid hormone and vitamin D metabolism or action
- Gastrointestinal hormones

## Indications and contraindications

Although it might be assumed that short children with CKD wish to be tall, the pros and cons of GH therapy, including the burden of receiving daily subcutaneous injections for many years, must be discussed with the patient and their family (Box 3). These considerations are of particular importance for immobilized patients and those with syndromic kidney diseases.

### Specific indicators for starting GH therapy

A height velocity below the twenty-fifth percentile for age and sex indicates progressive growth failure in a child presenting with short stature (that is, below the third percentile or with a height standard deviation score (SDS) below −1.88). Infancy is the most sensitive phase for the growth-suppressing effects of GH insensitivity. Any decrease in growth rate during this phase can result in severe growth retardation and a potentially irreversible loss of growth potential^45,46^. We therefore recommend that persistent growth failure, defined as height below the third percentile and height velocity below the twenty-fifth percentile beyond a period of 3 months in infants or 6 months in children and adolescents, be an indication for GH therapy once other potentially treatable risk factors for growth failure have been adequately addressed. Owing to the small size of available RCTs and a lack of long-term follow-up data from RCTs, the strength of this recommendation is graded as moderate.

Whether a height velocity below the twenty-fifth percentile is an indication to start GH even before height drops below the third percentile is unclear. Such early, preventive therapy might be more cost-effective than initiating GH therapy at an older age, when growth retardation has become evident and higher absolute GH doses are required to account for the higher body weight. The KDOQI guidelines for nutritional management in children with CKD suggest that GH should be initiated promptly if catch-up growth has not been induced within 3 months of initiating nutritional management^11^. Because a persistently reduced growth rate will ultimately result in short stature, we suggest that GH therapy is considered in children with height between the third and tenth percentile who have low height velocity (below the twenty-fifth percentile) that persists beyond 3 months in infants and beyond 6 months in children with growth potential provided that other potentially treatable risk factors for growth failure have been adequately addressed. However, no RCTs are available to support this approach; therefore, this recommendation is graded as weak.

### Benefits of GH in specific patient subgroups

#### Prepubertal patients

RCTs have demonstrated that GH stimulates growth in prepubertal children with pre-dialysis CKD, in patients on dialysis and after renal transplantation. A meta-analysis that included 16 RCTs and 809 patients^47^ showed that, compared with patients who received placebo or no additional treatment, patients in the intervention group who received 1 year of GH had a higher mean height SDS (difference of 0.91; 95% CI 0.58–1.23) and a higher mean height velocity (difference of 3.88 cm per year; 95% CI 3.32–4.44). Moreover, although reduced compared with levels in the first year of treatment, height velocity remained significantly greater in treated children than in untreated children during the second year of therapy (mean difference 2.3 cm per year; 95% CI 1.39–3.21).

To date, all except one RCT^48^ on GH treatment in children with pre-dialysis CKD have included only patients with CKD stage 3 or higher. Owing to insufficient data on the efficacy of GH in children with GFR above 60 ml/min/1.73 m^2^ and the weak effect of mildly decreased GFR on longitudinal growth, we do not recommend use of GH in short children with CKD stage 1 or 2 (ref.^49^) unless they have other conditions, such as nephropathic cystinosis, that predispose to poor growth^50^. Patients with cystinosis are prone to severe growth failure in early life despite a mild reduction in GFR due mainly to the presence of renal Fanconi syndrome. An uncontrolled study reported that GH treatment for up to 3 years increased growth rates in short children with nephropathic cystinosis compared with baseline, with superior growth responses observed in patients with early CKD (stage 1–3) compared with those with advanced CKD and on dialysis^50^.

#### Children before dialysis and on dialysis

Long-term follow-up of short-term RCTs and nonrandomized studies of GH treatment in prepubertal children with nondialysis-dependent CKD demonstrated that catch-up growth continues asymptotically over an extended treatment period, with reports of a cumulative increase in standardized height of 1.1–1.9 SDS within 5–6 years^51–53^. The growth response to GH treatment has been positively associated with residual renal function, target height, initial target height deficit and treatment duration and negatively associated with age at start of treatment^51^.

The response to GH treatment is significantly attenuated in children on dialysis compared with children with earlier stages of CKD, irrespective of dialysis modality, most likely owing to a higher degree of GH insensitivity^51,54^. However, the response to GH can be markedly improved by augmenting dialytic clearance with daily haemodialfiltration^55,56^. GH therapy also increases height in paediatric patients on dialysis irrespective of underlying bone histological features; bone formation rates are also higher in GH-treated patients than in controls owing to its osteoanabolic effects^57^. Although, renal transplantation does not uniformly result in catch-up growth in paediatric patients previously treated with GH, transplantation should not be withheld in these patients.

#### Paediatric renal transplant recipients

Persistent growth failure is common in children after renal transplantation as a result of reduced graft function and maintenance glucocorticoid (steroid) therapy^58,59^. A meta-analysis of five RCTs on growth outcome using steroid minimization protocols showed a significant increase in height SDS in the steroid avoidance group (mean difference of 0.38; 95% CI 0.07–0.68), particularly within the first year after steroid withdrawal (mean difference of 0.22; 95% CI 0.10–0.35), and in prepubertal patients (mean difference of 0.60; 95% CI 0.21–0.98)^60^.

Several RCTs have shown the benefit of GH therapy in short prepubertal renal transplant recipients. A meta-analysis of five prospective RCTs involving a total of 401 patients showed that patients receiving GH therapy had a significantly higher growth velocity 1 year after initiation of therapy than the control group, with a mean height SDS difference of 0.68 (95% CI 0.25–1.11)^61^. The mean difference in height SDS change between the treated and control group was 0.52 (95% CI 0.37–0.68), and the growth response to GH therapy was better in children younger than 10 years of age than in older patients^62^. We therefore recommend that GH therapy is considered for paediatric renal transplant recipients for whom expected catch-up growth cannot be achieved by steroid minimization or for patients in whom steroid withdrawal is not feasible owing to high immunological risk, particularly in children with suboptimal graft function (estimated GFR (eGFR) <50 ml/min/1.73 m^2^). We recommend that growth is monitored for at least 1 year post-transplantation before GH therapy is considered in order to allow for spontaneous catch-up growth.

#### Infants

In a study of 16 well-nourished infants with CKD stage 3–4, those randomly assigned to GH showed significantly higher length gains^28^. A second study of 12 infants treated with GH (8 of whom were on dialysis) reported similar rates of growth as in 15 matched controls with less severe CKD^63^. Data from the International Pediatric Peritoneal Dialysis Network (IPPN) registry showed that administration of GH was independently associated with improved length in infants and young children on chronic peritoneal dialysis^39^. Although the provision of adequate nutrition is certainly essential to promote the growth and development of infants with CKD, some infants show growth failure despite adequate caloric supply. Therefore, we recommend that early GH therapy is considered for infants with CKD stage 3–5 and those on dialysis to accelerate length and weight gain and to achieve the body size required for renal transplantation^64^. Given that the majority of infants included in the aforementioned studies were older than 3 months of age at the start of GH therapy as well as the need to assess spontaneous growth before GH treatment, we do not recommend GH therapy for infants younger than 6 months of age.

#### Pubertal patients

One study that followed GH-treated CKD patients from late prepubertal age to final height revealed that there was no overall effect of GH treatment on pubertal height gain^8^. By contrast, however, two studies of short pubertal transplant recipients reported that GH treatment initiated during puberty was associated with a higher mean gain in height than that of matched historical controls^65,66^. Likewise, an analysis of data from the Pfizer International Growth Database (KIGS) demonstrated that CKD patients who were either in early or late puberty at the time of GH therapy initiation experienced catch-up growth, with a significant cumulative increase in mean height SDS of 1.3 and 1.0 until near-adult height^67^.

### GH to improve adult height

Data on adult or near-adult height are available from ten nonrandomized trials and the KIGS, in which GH was administered for at least 2 years, comprising a total of 836 patients on various modes of renal replacement therapy. In five studies, a matched historical control group was included. The median change in standardized height until attainment of (near) adult height amounted to 1.1 SDS (range 0.2–1.6 SDS) in GH-treated patients (*P* < 0.05 for each final height measurement versus initial height measurement; Supplementary Table 4). This change corresponded to a median absolute increase in GH-treated patients of 7.4 cm (range 1.4–10.8 cm) in boys and 7.0 cm (range 1.3–10.1 cm) in girls on the basis of European reference values^19^. This calculation may represent a poor estimate (likely an underestimate) of median absolute height increase, because adult height was significantly lower in non-GH-treated controls than initial standardized height indices in all except one study^8,52,62,67–72^. Heights attained at the start of GH and throughout the duration of GH treatment were positively associated with final height, whereas time spent on dialysis, age at puberty onset and age of start of GH were negatively associated with final height^8,67^. Taken together, the available studies suggest that GH improves adult height in short prepubertal and pubertal CKD patients before and after renal transplantation.

### Contraindications

GH therapy improves height in paediatric dialysis patients, irrespective of the presence of underlying bone histological features, for example, high bone turnover due to secondary hyperparathyroidism^61^. However, severe secondary hyperparathyroidism (that is, characterized by intact PTH serum levels >500 pg/ml or increase ninefold above the normal range) is associated with diminished longitudinal growth and an increased risk of slipped capital femoral epiphysis in paediatric patients with CKD^73,74^. Because GH markedly stimulates bone growth, the risk of slipped capital femoral epiphysis might be further increased by GH treatment. Therefore, severe secondary hyperparathyroidism is a contraindication for GH therapy. Owing to its potential proliferative properties, GH is also contraindicated in patients with severe diabetic retinopathy or active malignancy. Finally, GH should not be used in patients with known hypersensitivity to the active substance or to any of the excipients (such as m-cresol) and in patients with acute critical illness suffering complications following heart surgery, abdominal surgery, multiple accidental trauma, acute respiratory failure or similar conditions^75^.

Box 3 Recommendations for assessing the indications and contraindications for GH treatment

- We recommend that pros and cons of growth hormone (GH) treatment are discussed with individual patients and their families before GH treatment is initiated. Such discussion is of particular importance for immobilized patients, and those with syndromic kidney diseases (grade X, moderate recommendation).
- We recommend that children with stage 3–5 chronic kidney disease (CKD) or on dialysis aged above 6 months should be candidates for GH therapy if they have persistent growth failure, defined as a height below the third percentile for age and sex and a height velocity below the twenty-fifth percentile, once other potentially treatable risk factors for growth failure have been adequately addressed and provided the child has growth potential (grade B, moderate recommendation).
- We recommend that GH therapy is considered for children with stage 3–5 CKD or on dialysis aged above 6 months who present with a height between the third and tenth percentile but persistent low height velocity (below the twenty-fifth percentile) once other potentially treatable risk factors for growth failure have been adequately addressed (grade D, weak recommendation).
- In children who have received a kidney transplant and have persistent growth failure, defined as a height below the third percentile for age and sex and a height velocity below the twenty-fifth percentile, we recommend initiating GH therapy 1 year after transplantation if spontaneous catch-up growth does not occur and steroid-free immunosuppression is not a feasible option (grade B, moderate recommendation).
- In children with CKD due to nephropathic cystinosis who have persistent growth failure, defined as a height below the third percentile for age and sex and a height velocity below the twenty-fifth percentile, we recommend that GH therapy is considered at all stages of CKD (grade C, moderate recommendation).
- GH therapy should not be started

- In patients with closed epiphyses (grade X, strong recommendation)
- In patients with known hypersensitivity to the active substance or to any of the excipients (grade X, strong recommendation)
- In the case of unwillingness of the patient or their family (grade X, strong recommendation)
- In patients with severe secondary hyperparathyroidism (parathyroid hormone >500 pg/ml) (grade X, moderate recommendation)
- In patients with proliferative or severe non-proliferative diabetic retinopathy (grade X, moderate recommendation)
- During the first year after renal transplantation (grade X, moderate recommendation)
- In patients with acute critical illness (grade X, strong recommendation)
- In patients with active malignancy (grade X, strong recommendation)

## Cost–benefit ratio of GH therapy

As outlined above, the expected increase in adult height after 2–5 years of GH treatment amounts to 7.2 cm (7.4 cm in boys and 7.0 cm in girls). According to our cost-effectiveness analysis (Supplementary Box 1; Supplementary Table 5), the corresponding total drug-related costs for a patient aged 5 or 12 years at start of treatment range from €13,000 to €37,900 and €27,100 to €80,100, respectively, depending on the length of treatment (2–5 years). The corresponding incremental cost per centimetre gained in adult height for a patient aged 5 or 12 years at the start of treatment ranges from €1,800 to €5,300 and from €3,800 to €11,100, respectively, again depending on the length of treatment (Supplementary Table 5). However, the actual cost of GH treatment might differ substantially among European countries. Our calculation was based on a cost of €22 per 1 mg GH, but the median country-specific costs range between €7 and €54 per 1 mg GH (Supplementary Table 6). The incremental cost-effectiveness of GH treatment obviously depends on the length of treatment and the required daily amount of GH, which is related to body weight; therefore, earlier initiation of GH therapy in a young patient would be more cost-effective if long-term dialysis is prevented by early renal transplantation. Finally, early initiation of GH treatment in a small child may substantially shorten the time taken to achieve the body size required for renal transplantation. We therefore recommend that the cost–benefit ratio is considered before initiating GH therapy in short children with CKD (Box 4).

Box 4 Recommendation for considering the cost–benefit ratio We suggest considering the cost–benefit ratio before initiating growth hormone treatment in short children with chronic kidney disease (grade D, weak recommendation).

## Schedule for GH therapy and monitoring

### GH dosage

The GH dosage used in the available RCTs and observational studies was 28–30 international units (IU)/m^2^ per week (equivalent to 0.045–0.05 mg/kg per day) by daily subcutaneous injections (Supplementary Tables 2–4). Twelve RCTs compared this dosage to placebo or no treatment^28,48,57,68,76–83^, 5 studies compared doses of 14 IU/m^2^ per week (equivalent to 0.023 mg/kg per day) to 28 IU/m^2^ per week^84–88^ and 1 study^65^ compared 28 IU/m^2^ per week to 56 IU/m^2^ per week (equivalent to 0.09 mg/kg per day). A 2012 meta-analysis demonstrated that compared with patients in the 14 IU/m^2^ per week group, patients in the 28 IU/m^2^ per week group experienced a 1.18 cm per year (95% CI 0.52–1.84) higher increase in height velocity and a 1.48 higher (95% CI 0.03–2.93) height velocity SDS after 1 year of treatment^47^. The one study that compared 28 IU/m^2^ per week with 56 IU/m^2^ per week reported no significant difference between the groups in the change in mean height SDS and mean height velocity. Therefore, we recommend that GH therapy is administered with a dosage of 0.045–0.05 mg/kg per day, irrespective of patients’ age, with dose adjustment according to body weight on regular intervals (Box 5).

### Frequency of GH administration

In healthy controls and in patients with GH deficiency, the bioavailability of GH after subcutaneous injection is approximately 80%, independent of sex. The T_max_ of GH is 3–6 hours and its half-life is 2–3 hours^89^. We recommend that GH is administered as daily subcutaneous injections because this was the mode of application used by all RCTs. To mimic the physiological circadian rhythm of endogenous GH secretion, evening injections are recommended. The injection side should be changed daily to avoid lipoatrophy.

### Type of GH

The available studies have used a variety of brands of GH^47^. In addition, GH biosimilars have been approved for the same indications as the reference product in Europe since 2006 following the completion of comprehensive comparability studies that assessed safety and efficacy^90^. Ongoing pharmacovigilance activities over the past 10 years have also not identified any relevant difference in safety profile. Therefore, we recommend both GH reference and GH biosimilar products for use in short children with CKD.

### Monitoring

Ongoing monitoring is necessary to evaluate the response to GH and to detect any potential adverse effects. The ultimate goal of GH treatment in children with CKD is to ‘normalize’ adult height. The therapeutic end point could be the attainment of the patient’s individual genetic target height or of a normal population-related adult height (that is, above the third percentile). Although the latter goal is certainly desirable, it may not be a feasible target if the patient’s genetic height potential is below the third percentile.

### Potential adverse events

The adverse effects of GH reported by RCTs are similar across different dosages and to those reported by participants in untreated control populations (Supplementary Tables 7, 8). However, the follow-up of RCTs to date has been relatively short; thus, monitoring the effect of long-term treatment requires assessment of registry data. A comprehensive comparison of the incidence of adverse events in large cohorts of paediatric CKD patients with and without GH treatment revealed no significant association between GH therapy and the incidence of malignancy, slipped capital femoral epiphysis, avascular necrosis, glucose intolerance, pancreatitis, rapid progression of CKD, acute allograft rejection or fluid retention^91^. Commonly reported adverse effects are discussed below.

#### Intracranial hypertension

An analysis of registry data from the Genentech National Cooperative Growth Study showed that intracranial hypertension was reported in 3 of 1,376 CKD patients^44^. Although in all three patients intracranial hypertension occurred after discontinuation of GH, we recommend a baseline fundoscopy before initiation of GH and, in the presence of persistent headache or vomiting, an immediate work-up including fundoscopy should be performed.

#### Glucose intolerance

Although insulin secretion increases during the first year of GH treatment and hyperinsulinaemia persists during long-term therapy, evidence suggests that GH treatment for ≤5 years does not typically have an adverse effect on glucose tolerance^92^. The incidence of obesity in CKD patients has markedly increased over the past decade, and close monitoring of glucose metabolism is advised when initiating GH therapy in these patients owing to an increased risk of impaired glucose tolerance. Patients with nephropathic cystinosis have an increased risk of diabetes mellitus, irrespective of GH treatment^93^.

#### Secondary hyperparathyroidism and orthopaedic complications

Aggravation of secondary hyperparathyroidism has been reported as an occasional complication of GH therapy. GH might have a direct stimulatory effect on the parathyroid gland or might have subtle effects on calcium and phosphate homeostasis, which in turn stimulate PTH secretion^57,74^. In addition, increased longitudinal bone growth by GH treatment might unmask pre-existing renal osteodystrophy^57^. Therefore, CKD-MBD should be adequately treated according to current guidelines before initiation of GH therapy^94^. GH therapy should be withheld in patients with persistent severe secondary hyperparathyroidism (PTH >500 pg/ml) and can be reinstituted when PTH levels return to the desired target range^35–37^. In addition, rapid growth might contribute to an increased risk of slipped capital femoral epiphysis. We therefore recommend obtaining bone radiographs if symptoms occur and to discontinuing GH therapy if a diagnosis of slipped capital femoral epiphysis is confirmed.

#### Renal function

RCTs have not shown adverse effects of GH on renal function in paediatric patients with nondialysis CKD^47^. Likewise, an analysis of two large cohort studies did not show evidence of accelerated eGFR decline in children with CKD treated with GH compared with untreated patients over 10 years of follow-up^95^. However, because anecdotal reports of increases in serum creatinine level exist, we recommend close monitoring of renal function according to the stage of CKD, for example, monitoring in 3-month intervals in patients with CKD stage 3–4. GH therapy may need to be stopped if an unexplained decrease in eGFR occurs.

#### Bone and sexual maturation

No evidence exists to support an association between advanced pubertal growth spurts or accelerated bone age maturation with GH therapy^8,47^. However, anecdotal reports of accelerated pubertal development with GH therapy exist; therefore, yearly assessment of pubertal stages according to the Tanner stages is recommended for children older than 10 years of age. In addition, yearly assessment of bone age by radiography of the left wrist is suggested to exclude accelerated bone maturation. Discontinuation of GH treatment should be considered for patients with evidence of accelerated bone maturation.

### Safety aspects of GH therapy in transplanted patients

Registry data and post-marketing surveillance by GH manufacturers have revealed no increased risk of malignancy in paediatric renal transplant recipients treated with GH^96–98^. RCTs have also not reported differences in the rate of rejection episodes (risk ratio 1.56; 95% CI 0.97–2.53) or eGFR between treatment groups^61^.

### Identification and management of non-responders

An adequate growth response can be assumed if height velocity during the first year of GH treatment is greater than 2 cm per year over baseline. Such an increase would be approximately half of that reported in RCTs (mean increase 3.88 cm per year; 95% CI 3.32–4.44)^47^. For patients who do not adequately respond to GH therapy, we recommend assessment of patient adherence to GH therapy, including measurement of serum IGF1 levels, weight-adjusted GH dosage and assessment of nutritional and metabolic factors, as recommended before initiation of GH therapy^9^. As outlined above, higher GH doses are not more efficient than lower doses; therefore, higher GH doses cannot be recommended in non-responders.

### Discontinuation of GH therapy

Termination of GH therapy should be considered in patients who do not adequately respond to GH treatment for a period of at least 6 months. Syndromic kidney disease might be one reason for a poor response to GH treatment. However, non-adherence to daily subcutaneous injections or inadequate GH dosing for the individual’s weight should be excluded. CKD-associated growth-limiting factors (see above) should also be adequately addressed in all non-responders, and GH may be transiently stopped to address these issues. GH treatment must be stopped in all patients at the time of renal transplantation. It seems to be reasonable to stop GH when the patient has attained his or her genetic target height. In addition, GH may be stopped when a patient reaches his or her genetic target height percentile. However, in a study of 22 patients who had a pause in GH therapy after attainment of target height percentile, withdrawal of GH led to maintenance of height SDS in only 27% of patients and a marked reduction in growth velocity in 73% of patients, who subsequently required reinstitution of GH therapy^99^. Alternatively, the dose of GH may be reduced when a patient reaches their genetic target height percentile, but the outcomes associated with this approach have not been systematically studied.

In late-pubertal adolescents, if height velocity drops below 2 cm per year and/or epiphyseal growth plate closure is evident on radiography, GH should be stopped as no further growth potential can be expected. Stopping GH in the case of severe adverse effects, a severe illness or trauma is recommended^100–102^.

Box 5 Recommendations for GH treatment and monitoring

- We recommend that growth hormone (GH) is given at a dose of 0.045–0.05 mg/kg body weight per day by subcutaneous injections in the evening (grade B, moderate recommendation).
- We suggest that parents and physicians encourage children from about 8–10 years of age to do the GH injections on their own if adequate training and adherence is ensured (grade D, weak recommendation).
- We recommend both GH reference and GH biosimilar products for use in short children with chronic kidney disease (CKD) (grade B, moderate recommendation).
- We suggest clinic visits every 3–6 months or more frequently for young patients and those with advanced CKD to monitor stature, height velocity, pubertal development, skeletal maturation on wrist radiography, renal function, thyroid hormone levels (TSH and free T3), serum glucose, calcium, phosphate, bicarbonate and parathyroid hormone levels (grade D, weak recommendation).
- If height velocity in the first year of GH treatment is less than 2 cm per year over baseline, we recommend assessment of patient adherence to GH therapy, including measurement of serum insulin-like growth factor 1 levels, weight-adjusted GH dosage and assessment of nutritional and metabolic factors, as recommended before initiation of GH therapy (grade B, moderate recommendation).
- We recommend stopping GH

- When epiphyseal closure is demonstrated (grade X, strong recommendation)
- At the time of renal transplantation (grade X, strong recommendation)
- In patients with persistent severe secondary hyperparathyroidism (parathyroid hormone (PTH) >500 pg/ml). GH may be reinstituted when levels return to the desired PTH target range (grade X, moderate recommendation)
- With occurrence of intracranial hypertension (grade X, strong recommendation)
- In patients with slipped capital femoral epiphysis (grade X, strong recommendation)
- If the patient does not adequately respond to GH treatment despite optimal nutritional and metabolic control (grade X, moderate recommendation)
- In patients with accelerated bone maturation (grade X, moderate recommendation)
- In case of an unexplained decrease in estimated glomerular filtration rate (grade X, moderate recommendation)
  - We suggest that cessation of GH treatment is considered

- When the patient reaches his or her genetic target height percentile. GH may be reinstituted if catch-down growth occurs (grade X, moderate recommendation)
- When the patient reaches his or her genetic target height (grade X, moderate recommendation)

## Implementation and audit recommendations

The CPRs reported here will be disseminated widely through teaching lectures and webinars at an international level (for example, through International Pediatric Nephrology Association (IPNA) teaching courses and webinars), at the European level (for example, through the annual ESPN precongress teaching course, ESPN-IPNA master classes and webinars) and at an individual country level (for example, through annual National Pediatric Nephrology Congresses). The ESPN CKD-MBD, Dialysis and Transplantation working groups will audit the effectiveness and safety of the recommendations. To achieve this aim, growth outcome in children with CKD on GH treatment will be measured and reported in a European patient registry. The audit outcomes will be published and recommendations updated as necessary.

## Research recommendations

Several areas of study have the potential to guide future evidence-based recommendations for GH therapy in children with CKD on dialysis and after renal transplantation. For example, animal studies suggest additive effects of treatment with recombinant IGF1 in combination with GH^103^. This combination approach should therefore be tested in short children on dialysis in whom growth failure is often incompletely resolved by GH treatment.

Animal studies also suggest that linear bone growth in CKD is adversely affected by the increased presence of inflammatory cytokines, which activate the suppressor of cytokine signalling 2 (SOCS2) pathway^104^. Future studies should therefore address the growth-promoting effects of measures targeting the SOCS2 pathway in short children with CKD.

One study showed that pharmacological inhibition of epiphyseal growth plate closure with the aromatase inhibitor anastrozole extended the duration of the growth potential period and increased adult height in short male adolescents treated with GH^105^. This approach should therefore be tested in severely growth-restricted adolescents on long-term dialysis because these patients are prone to persistent growth failure despite GH treatment^51,54^.

A study of the intra-individual variations in body proportions (for example, the ratio between trunk length and leg length) before and after GH treatment is warranted (because CKD is associated with disproportionate short stature) to identify how GH treatment affects different aspects of growth^106^.

Further research is also needed into the effect of GH treatment before renal transplantation on growth after renal transplantation, precision (personalized) medicine approaches to better provide individualized approaches to therapeutic management and the development of lower cost biosimilars with the need for less frequent injections or even oral preparations.

## Conclusions and perspectives

Short stature is a frequent complication in children with chronically impaired renal function (typically defined as <50% of normal function), even after renal transplantation. We recommend that children with stage 3–5 CKD or those on dialysis older than 6 months of age should be candidates for GH therapy if they have persistent growth failure (defined as a height below the third percentile for age and sex and a height velocity below the twenty-fifth percentile) once other potentially treatable risk factors for growth failure have been adequately addressed and provided the child has growth potential. In children who have received a kidney transplant and fulfil the above growth criteria, we recommend initiating GH therapy 1 year after transplantation if spontaneous catch-up growth does not occur and steroid-free immunosuppression is not a feasible option. GH is given by daily subcutaneous injections, usually for a duration of several years until the patient reaches their final height or until renal transplantation. The expected increase in final height after 2–5 years of GH treatment amounts to approximately 7.2 cm. Patients and their families should be informed before initiation of GH therapy that individual growth responses differ widely. The GH injections usually do not hurt, and school-aged children may do the injections on their own if adequate training and adherence are ensured. Adverse effects of GH treatment are rare and include glucose intolerance and increased cranial pressure. The benefits of GH treatment with respect to increased stature must be weighed against the burden of daily subcutaneous injections on an individual basis (Boxes 1,3–5; Supplementary Table 9).

## Supplementary information

Supplementary Information

## Glossary

Somatomedin: Somatomedins are a group of proteins, such as insulin-like growth factor 1 (IGF1), that are predominantly produced by the liver and mediate the growth-promoting effects of growth hormone at the growth plate level.
Epiphysis: The rounded end of a long bone at its joint with an adjacent bone or bones. Between the epiphysis and the diaphysis (the long midsection of the long bone) lies the metaphysis and the epiphyseal plate (growth plate). As a child grows, the epiphyses become calcified and appear on radiography images. As growth nears conclusion, bones begin to approach the size and shape of adult bones. The remaining cartilaginous portions of the epiphyses become thinner. As these cartilaginous zones become obliterated, the epiphyses are said to be ‘closed‘ and no further lengthening of the bones will occur.
Tanner–Whitehouse Mark II method: A method used to estimate the expected adult height of children using a linear model of height and bone age. The latter is assessed by the Tanner–Whitehouse II method, which is a bone-specific scoring technique in which a numerical score is assigned to selected wrist bones on radiography of the left wrist depending on the appearance of certain well-defined maturity indicators.
Gonadotropin-releasing hormone: (GnRH). A releasing hormone responsible for the release of follicle-stimulating hormone and luteinizing hormone from the anterior pituitary. GnRH constitutes the initial step in the hypothalamic–pituitary–gonadal axis.
Luteinizing hormone: (LH). A sex hormone produced by gonadotropic cells in the anterior pituitary gland. In females, an acute rise of LH triggers ovulation and the development of the corpus luteum, which is a temporary structure in female ovaries. In males, LH stimulates production of testosterone in the testicle.
Fundoscopic examination: A test that enables a health professional to see inside the fundus of the eye and other structures using an ophthalmoscope. It is used to detect papilledema.
Papilledema: Swelling of the optic disc (the point of exit of the optic nerve in the eye) that is caused by increased intracranial pressure due to many causes including medications such as growth hormone. Vision loss can result if the underlying condition is not treated.
Bone age: The degree of maturation of a child’s bones as seen by radiography of the left wrist. The bone age of a child is the average age at which children reach this stage of bone maturation. Usually, bone age is the same as the biological age. An advanced or delayed bone age indicates a reduced or increased growth potential, respectively, compared with children with the same age and height.
Tanner stages: A scale of physical development in children. The scale defines physical measurements of development on the basis of external primary and secondary sex characteristics, such as the size of the breasts and genitals, testicular volume and the development of pubic hair.
Syndromic kidney diseases: Kidney diseases that also involve other organs, including the brain, bone and endocrine system.
Height standard deviation score: (SDS). A measure of height that expresses the anthropometric value as the number of standard deviations below or above the reference mean height of healthy children. A score below –1.88 indicates short stature, which corresponds to a height below the third percentile of healthy children of the same age and sex.
Near-adult height: A stage at which no relevant further height gain can be expected. It is usually defined as a height velocity less than 1 cm per year in the presence of advanced clinical signs of puberty in adolescents.
Slipped capital femoral epiphysis: A fracture through the growth plate (physis) that results in slippage of the overlying end of the femur (metaphysis). It is the most common hip disorder in adolescence. Risk factors include childhood obesity and treatment with growth hormone.
T_max_: The time it takes a drug such as human growth hormone to reach the maximum concentration (C_max_) in blood after injection.
Lipoatrophy: Localized loss of fat tissue, which may occur as a result of subcutaneous injections of insulin or human growth hormone, especially when the same injection site is used.
Genetic target height percentile: The genetic potential for height (genetic target height) is commonly estimated from parental height according to the Tanner method. The genetic height percentile is estimated by plotting the value of an individual’s genetic target height on a reference chart of healthy children.
