# Supplementary material for: Clinical practice recommendations for growth hormone treatment in children with chronic kidney disease
Source: Nat Rev Nephrol. 2019 Jun 13;15(9):577–89. doi: 10.1038/s41581-019-0161-4 (PMC7136166; doi:10.1038/s41581-019-0161-4)
Supplement: Supplementary file 1 — Supplementary Information [file 41581_2019_161_MOESM1_ESM.pdf]

nature REVIEWS NEPHROLOGY

Clinical practice recommendations for growth hormone treatment in children with chronic kidney disease

Jens Drube, Mandy Wan, Marjolein Bonthuis, Elke Wühl, Justine Bacchetta, Fernando Santos, Ryszard Grenda, Alberto Edefonti, Jerome Harambat, Rukshana Shroff, Burkhard Tönshoff and Dieter Haffner, on behalf of the European Society for Paediatric Nephrology Chronic Kidney Disease Mineral and Bone Disorders, Dialysis, and Transplantation Working Groups

https://doi.org/10.1038/s41581-019-0161-4

Supplementary Box 1 | Cost-effectiveness analysis

Most pediatric CKD patients have advanced CKD at the time of initiation of GH treatment, and will undergo renal transplantation when GH treatment is terminated. The duration of GH treatment is mainly determined by the age at onset of CKD, its rate of progression and the availability of a renal transplant. Therefore, our cost-effectiveness analysis included two hypothetical scenarios: (i) case 1, a child with early-onset CKD requiring GH therapy at the age of 5 years, (ii) case 2, an adolescent with late onset or slowly progressive CKD requiring GH treatment at the age of 12 years. The mean duration of GH treatment in studies reporting on final height ranged between 2 and 5 years; therefore, we used this range to estimate the cumulative drug-related costs. In addition, the estimates for drug dose were based on daily GH doses of 0.045 mg/kg body weight and were calculated for the respective age- and sex-related 25th weight percentile using the WHO reference data, assuming that patients will have a height below the 3rd percentile at the time of initiation of GH treatment and will show catch-up growth into the lower normal range thereafter (**Supplementary Table 5**) [S1].

Since the costs for patient monitoring are less than 3% of total treatment-related costs, only drug-related costs were taken into account for this analysis [S2]. The cost for GH differs considerably among European countries, and a price of €22 per 1 mg GH, based on the median cost in eight representative European countries, was used (**Supplementary Table 6**).

In clinical studies the height standard deviation score (height SDS) is often used to compare growth in children differing in age and sex. Height SDS is a conversion of height (or length) that represents the number of standard deviations (SD) from the mean height for age and sex. A child with a height SDS less than -1.88, which corresponds to the 3rd percentile, has short stature. Therefore, the mean increase in final height in GH treated patients was calculated as the difference between standardized final height (height SDS) and standardized height at the start of GH therapy for all available studies reporting on adult height with treatment periods of at least 2 years (**Supplementary Table 4**). The median increase in standardized height in these studies (1.1 SDS) was converted to cm (7.4 cm in boys, 7.0 cm in girls) by use of European reference values. Thus, an expected gain in final height of 7.2 cm was used in the cost-effectiveness analysis; that is, a calculation of the incremental cost per centimeter gained in final height [S3].

Supplementary Table 1 | Inclusion and exclusion criteria used in 18 randomized clinical trials (RCTs) of GH treatment in children with chronic kidney disease.

Ref.	Major inclusion criteria	Major exclusion criteria
Bacchetta et al. (2013)<br>[S4]	• CKD stage 5D<br>• Age 2–21 yrs.<br>• No auxological inclusion criteria given	• Poor medical adherence<br>• Parathyroidectomy<br>• Epiphyseal growth plate closure<br>• Treatment with prednisone or any other immunosuppressive agent
Santos et al. (2010)<br>[S5]	• CKD stage 3–5D<br>• Well-nourished<br>• Age 12 ± 3 months<br>• Length < –2.0 SDS and HV < 50th percentile	• Non-CKD related hormonal, genetic, neurologic, osseous conditions<br>• Suspected allergy to the trial product<br>• Treatment with corticosteroids<br>• Inadequate metabolic control of CKD (severe sHPT, acidosis, sodium or water deficits)
Hertel et al. (2002)<br>[S6]	• CKD (eGFR <40 ml/min/1.73 m^2 or on dialysis)<br>• Age 3–18 years<br>• BA <12 yrs (girls), < 10 yrs (boys)<br>• Height < –2.0 SDS and HV velocity SDS <0.0	• Abnormal thyroid status<br>• Endocrine or metabolic disease other than sHPT<br>• Growth retardation due to failure of other organs, or psychosocial dwarfism
Fine et al. (2002)<br>[S7]	• CKD stage 5T<br>• Height < –2.0 SDS<br>• BA < 15 yrs (girls), < 16 yrs (boys)	• Specific cause for the growth retardation other than those implicated in renal allograft recipients<br>• Active malignancy or treated for a malignancy within one year<br>• Diabetes mellitus<br>• Gonadotropin deficiency on estrogen/androgen therapy<br>• Deformities obviating accurate height measurements<br>• Other investigational drug within 6 months of the study
Sanchez et al.(2002)<br>[S8]	• CKD stage 5T<br>• Prepubertal<br>• Normal bone formation rate or adynamic bone disease on bone histomorphometric analysis<br>• No auxological inclusion criteria given	• Willingness to undergo bone biopsy procedure<br>• Histological evidence of sHPT
Kuizon et al. (1998)<br>[S9]	• CKD stage 5D on PD<br>• No auxological inclusion criteria given	• Not given
Maxwell et al., (1998)<br>[S10]	• CKD stage 5T<br>• At least 1 year after KTx<br>• eGFR >20 ml/min/1.73m^2<br>• Height <3rd percentile or HV <25th percentile<br>• Normal thyroid function	• Height velocity > 75th percentile during the preceding 6 months<br>• Treatment with any form of GH in the past year<br>• Previous malignancy<br>• Severe congenital abnormality<br>• Diabetes mellitus<br>• Uncontrolled renal bone disease
Powell et al. ,(1997)<br>[S11]	• CKD (eGFR >5 and <75 ml/min/1.73 m^2)<br>• Age > 2.5 years<br>• Ability to stand for height measurement<br>• BA < 10 yrs (girls), < 11 yrs. (boys)<br>• Prepubertal	• Serum albumin <2.5 g/dl<br>• Medications which influence growth<br>• Presence of illnesses affecting growth<br>• Diabetes mellitus<br>• Present or past history of malignancy
Ito et al. (1997)	• CKD (eGFR <40 ml/min/1.73 m^2 or on	• Not given

[S12]	dialysis)<br>• BA < 12 yrs (girls), < 13 yrs (boys)<br>• Prepubertal<br>• Height or HV < –2.5 SDS	
Kitagawa et al. (1997) [S13]	• CKD (eGFR < 40 ml/min/1.73 m^2)<br>• BA < 12 yrs (girls), < 13 yrs (boys)<br>• Prepubertal<br>• Height or HV < –2.5 SDS	• Not given
Broyer et al. (1996) [S14]	• CKD stage 5T<br>• Prepubertal	• Not given
Kawaguchi et al. (1996 [S15]	• CKD (eGFR < 40 ml/min/1.73 m^2 or on dialysis)<br>• BA < 12 yrs (girls), < 13 yrs (boys)<br>• Prepubertal<br>• Height or HV < –2.5 SDS<br>• CKD stage 5T (eGFR > 30 ml/min/1.73 m^2)<br>• BA <13 yrs (girls), <14 yrs (boys)<br>• Prepubertal or early pubertal	• Not given
Hokken-Koelega et al. (1996) [S16]	• CKD stage 5T<br>• Height < –1.88 SDS and HV < 25th percentile<br>• Prepubertal<br>• BA < 10 yrs (girls), < 12 yrs (boys)	• Thyroid dysfunction<br>• Metabolic acidosis<br>• Previous sex hormone treatment<br>• Growth retardation due to other causes
Fine et al. (1995) [S17]	• CKD (eGFR > 5 and < 75 ml/min/1.73 m^2)<br>• Age < 2.5 yrs<br>• BA <10 yrs (girls), < 11 yrs (boys)<br>• Prepubertal<br>• Height < 3rd percentile	• Specific cause of growth retardation other than CKD<br>• Inability to obtain accurate height measurements (e.g. severe scoliosis, meningomyelocele)<br>• Medications that influence growth<br>• Diabetes mellitus<br>• Active malignancy or treated for a malignancy within the past year
Hokken-Koelega et al. (1994) [S18]	• CKD (eGFR < 20 ml/ min/1.73 m^2 or on dialysis<br>• Height < –1.88 SDS and HV < 50th percentile<br>or<br>• Height < 0.0 SDS and HV < 25th percentile<br>• Prepubertal<br>• BA <10 yrs (girls), <12 yrs (boys)	• Specific cause of growth retardation other than CKD<br>• Hypothyroidism<br>• Metabolic acidosis<br>• Clinical or radiographic signs of osteodystrophy<br>• No previous treatment with anabolic or sex steroids
Hokken-Koelega et al. (1994) [S19]	• > 12 months after KTx<br>• > 6 months no history of rejections<br>• Height < –1.88 SDS and HV < 50th percentile<br>or<br>• Height <0.0 SDS and HV < 25th percentile<br>• Prednisone dosage <0–0.25 mg per kg per day or 0.50 mg per kg every other day for 6 months<br>• BA <8 yrs (girls), <10 yrs (boys)	• Specific cause of growth retardation other than CKD<br>• Hypothyroidism<br>• Metabolic acidosis<br>• No previous treatment with anabolic or sex steroids
Fine et al.(1994) [S20]	• CKD (eGFR > 5 and < 75 ml/min/1.73 m^2)<br>• Prepubertal,	• Specific cause of growth retardation other than CKD<br>• Inability to obtain accurate height

	• BA <10 yrs (girls), <11 yrs (boys)<br>• Height < 3rd percentile	measurements<br>• Corticosteroids or other medications than influence growth<br>• Diabetes mellitus<br>• Active malignancy or treated for a malignancy within the past year<br>• Other investigational drug within 2 months of assignment into the study
Hokken-Koelega et al.(1991) [S21]	• CKD (eGFR < 20 ml/ min/1.73 m^2 or on dialysis)<br>• Height < -1.88 SDS and HV < 25th percentile<br>• Prepubertal<br>• BA < 10 yrs (girls), < 12 yrs (boys)	• Specific cause of growth retardation other than CKD<br>• Hypothyroidism<br>• Metabolic acidosis<br>• Clinical or radiographic signs of osteodystrophy<br>• Previous treatment with anabolic or sex steroids

BA, bone age; CKD, chronic kidney disease; eGFR, estimated glomerular filtration rate; GH, growth hormone; KTx, kidney transplantation; HV, height velocity; SDS, SD score; sHPT, secondary hyperparathyroidism.

Supplementary Table 2 | 18 RCTs and a meta-analysis on GH therapy in children with CKD included in the systematic review

Ref. (country of origin)	Study design	Patients	Intervention and comparator	Outcomes
Bacchetta et al. (2013) [S4] (USA)	Single center, RCT, open labeled	33 patients with CKD stage 5D (all on PD)<br><br>GH: n=15 (M/F 6/9), prepubertal: 8/15, low–normal bone turnover n=7, high bone turnover n=8<br><br>Controls: n=18 (M/F 9/9), prepubertal 9/18, low–normal bone turnover n=7, high bone turnover n=11	GH<br>0.05 µg/kg/d s.c.<br><br>Controls:<br>No GH<br><br>In addition, patients with high bone turnover received 1µg calcitriol thrice weekly	Delta height SDS, GH vs. no-GH 0.32±0.08 (*P*<0.01). Bone formation rate increased in patients with low bone turnover and normalized (decreased) in patients with high bone turnover receiving GH therapy (each *P* <0.05)
Hodson et al. (2012) [S22] (Australia)	Cochrane Review comprising: 16 RCTs<br><br>including 10 RCTs	n=809 (CT, dialysis, KTx)<br><br>n=560 (CT, dialysis, KTx); most patients prepubertal or early pubertal	GH, 28 IU/m² per wk daily vs. placebo or no treatment	Delta height SDS at 1 year: 0.82 (95% CI 0.56–1.07) independent of pubertal status and CKD stage<br><br>Increase in HV: at 6 months, 2.85 cm/6 mo (95% CI 2.22–3.84); at 12 months, 3.88 cm/yr (95% CI 3.32–4.44)<br><br>HV greater by 2.3 cm/yr (95% CI 1.39–3.21) than in controls during the 2nd treatment year<br><br>HV in higher GH dose group exceed that in the lower GH dose by 1.18 cm/yr (95% CI 0.52–1.84)<br><br>The frequency of reported adverse effects of GH was generally similar to that in control groups
	5 RCTs	n=231 (CT, dialysis); most patients prepubertal	GH, 28 vs. 14 IU/m² per wk daily	
	1 RCT	n=16 (KTx), prepubertal	GH, 56 vs. 28 IU/m² per wk daily	
Santos et al. (2010) [S5] (Spain)	Multicenter, RCT, open labeled	n=16 (M/F 13/3, CKD stage 3-5D; 3 on PD) Age 12 ± 3 months<br><br>GH: n=8<br><br>Controls: n=8	GH:<br>0.33 mg/kg/wk for 12 months<br><br>Controls:<br>No GH treatment	Length gain in infants treated with GH was higher (*P*<0.05) than in controls (HV, 14.5 versus 9.5 cm/yr; change in height SDS, 1.43 versus -0.11); GH treatment increased forearm bone mass and serum concentrations of total and free IGF-I and IGFBP-3
Hertel et al. (2002) [S6] (Denmark)	Multicenter, RCT, open labeled	CKD stage 3-5D<br><br>GH 1: n=14 (M/F 12/2)<br><br>GH 2: n=15 (M/F	GH 1: 28 IU/m²/wk daily<br><br>GH 2: 14 IU/m²/wk daily	HV SDS was increased to 3.0 SDS in the 1st year in the low-dose, and to 3.8 SDS in the high-dose group (each *P*<0.05). In the 2nd year, HV SDS was increased to 1.3 SDS in the

		12/3)		low-dose group and to 2.1 SDS in high-dose group (each *P*<0.05).
Fine et al.<br>(2002) [S7]<br>(USA)	Multicenter,<br>RCT, open<br>labeled	n=68 (KTx)<br><br>GH:<br>n=36 (M/F 29/7);<br>Tanner stage I n= 26,<br>Tanner stage 2–3<br>n=10<br><br>Controls;<br>n=27 (M/F 25/2);<br>Tanner stage 1 n=14,<br>Tanner stage 2–3<br>n=13	GH:<br>0.05 mg/kg/d<br>for 12 months<br><br>Controls:<br>No GH treatment	Delta height SDS, 0.49 vs. –0.10 (*P*<0.001);<br>no increased rejection rates on GH; previous rejection was predictive for future rejections on GH teatment; adverse events similar
Sanchez et al.<br>(2002) [S8]<br>(USA)	Single center,<br>RCT, open<br>labeled	n=21 (KTx)<br><br>GH: n=11<br><br>Controls: n=10	GH: 28 IU/m²/wk for 24 months<br><br>Controls: no GH treatment	GH: height SDS increased from -2.0±1.1 to –1.1±1.0 (*P*<0.02) after 12 months. Controls: no significant change; height velocity was greater in GH group versus controls (8.0±2.1 cm/year vs. 4.8±1.7 cm/year, *P*<0.01)
Kuizon et al.<br>(1998) [S9]<br>(USA)	Multicenter,<br>RCT, open<br>labeled	n=14 (CKD stage 5D; all on PD)<br><br>GH: n=6<br><br>Control: n=8	GH: 28 IU/m²/wk daily<br><br>Control: no GH treatment	Height SDS after 1 year higher in GH group (–1.4 ± 0.6) compared to controls (–2.2 ± 1.1; *P*<0.05)
Maxwell et al.<br>(1998)<br>[S10] (UK)	Multicenter<br>RCT, open<br>labeled	n=22, CKD stage 5T;<br>M/F 18/4<br><br>GH: n=13<br>(prepubertal n=9,<br>pubertal n=4)<br><br>Controls: n=9<br>(prepubertal n=6,<br>pubertal n=3)	GH: 0.05 mg/kg/d (equivalent to 4 IU/m^2/d) in the 1st yr<br><br>Control: no GH treatment in the 1st yr<br><br>All patients received GH in the 2nd yr	1st yr: mean (SE)<br>Prepubertal: HV and change in height SDS was significantly higher in GH group vs. controls (8.1 (0.9) vs. 3.7 (0.6) cm/year and 0.6 (0.1) vs. –0.3 (0.2; each *P*< 0.001).<br>Pubertal: HV and delta height SDS were higher in GH vs. controls (10.1 (0.6) vs. 3.9 (1.3) cm/year and 0.6 (0.1) vs.–0.1 (0.2); each *P*<0.05)
Powell et al.<br>(1997) [S11]<br>(USA)	Multicenter,<br>RCT, open<br>labeled	n=44; CKD stage 3–5D; prepubertal 44/44<br><br>GH: n=30 (M/F 25/5); age 5.6 ± 2.0<br><br>Controls: n=14 (M/F 12/2); age 5.7 ± 2.6	GH: 0.05 mg/kg/d for 12 months<br><br>Controls: no GH treatment	GH: HV, 9.1 ± 2.8 cm; weight gain, 3.5 ± 1.5 kg<br><br>Controls: HV, 5.5 ± 1.9 cm; weight gain, 2.2 ± 1.0 kg<br>(each *P*<0.01 GH vs. controls)
Ito et al.<br>(1997) [S12]<br>(Japan)	Multicenter,<br>RCT, open<br>labeled	n=29 (M/F 5/24); CKD stage 3–5 n=21; dialysis n= 8<br><br>Prepubertal n=26<br>Tanner stage 2 n=2<br>Tanner stage 3 n=1	2 IU/m²/d vs.<br>4 IU/m²/d for 12 months.<br>Thereafter, 4 IU/m²/d for 12 months	Significant increase in height SDS, HV and HV SDS in GH group compared to pre-treatment (each *P*<0.05); no significant difference between different treatment groups
Kitagawa et al.<br>(1997) [S13]<br>(Japan)	Multicenter<br>RCT, open<br>labeled	CKD stage 5D, prepubertal<br><br>GH 0.5:<br>n=26 (M/F 15/11)	GH 0.5: 0.5 IU/kg/wk daily for 24 months<br><br>GH 1.0: 1.0	Height SDS:<br>GH 0.5: no significant increase during the 1st yr.<br>GH 1.0: significant increase during the 1st yr (*P*<0.05).

		GH 1.0:<br>n=28 (M/F 17/11)	IU/kg/wk daily for 24 months	HV:<br>GH 0.5: no significant increase.<br>GH 1.0: significant increase during the 1st and 2nd yr compared to baseline (each *P*<0.01).<br>HV SDS:<br>In both groups significant increase during the 1st and 2nd yr compared to baseline (each *P*<0.01)
		CKD 3–5D, prepubertal<br><br>GH 0.5:<br>n=28 (M/F 19/9)<br><br>GH 1.0:<br>n=30 (M/F 22/8)	GH 0.5: 0.5 U/kg/wk daily for 24 months<br><br>GH 1.0: 1.0 U/kg/wk daily for 24 months	Height SDS: Significant increase in both groups (each *P*<0.05).<br>HV: Significant increase in both groups (each *P*<0.05).<br>HV SDS: In both groups significant increase during the 1st and 2nd yr compared to baseline (each *P* <0.01)
Broyer et al. (1996) [S14] (France)	Multicenter RCT, open labeled	GH: n=106 (n=67 prepubertal, M/F 71/35, KTx 106/106)<br><br>Control: n= 97 (n=51 prepubertal M/F 72/25, KTx 97/97)	GH:<br>30 IU/m²/wk daily<br><br>Control: no GH treatment	Change in HV and delta height SDS during the 1st yr higher in GH group vs. controls (each *P*<0.0001).<br>2nd yr, HV remained greater in GH group compared to baseline resulting in further increase in height SDS
Kawaguchi et al.<br>(1996) [S15] (Japan)	Multicenter, RCT, open labeled	n=83; CKD stage 3–5D/T (including 23 KTx patients)<br><br>GH 0.5: n=54 (CKD stage 3–5D n=28; M/F 34/20)<br><br>GH 1.0: n=58 (CKD stage 3–5D n=30; M/F 39/19)	GH 0.5: 0.5 IU/kg/wk for 12 months<br><br>GH 1.0: 1.0 IU/kg/wk for 12 months	CKD stage 3–5: HV significantly increased in both groups, and was higher in GH 1.0 vs. GH 0.5 (each *P*<0.01).<br>CKD stage 5D: HV significantly increased in both groups, and was higher in GH 1.0 vs. GH 0.5 (each *P*<0.01).<br>CKD stage 5T: HV increased increased in both groups (each *P*<0.05) and did not differ between groups; 7/23 patients showed acute rejection episodes.
Hokken-Koelega et al. (1996) [S16] (Netherlands)	Multicenter, RCT, open labeled	n=11; CKD stage 5T; prepubertal<br><br>GH: n=6 (M/F 4/2)<br><br>Controls: n=5 (M/F 4/1)	GH: 28 IU/m²/wk daily for 6 months<br><br>Controls: placebo	HV exceeded that of placebo by 2.9 cm/6 months; no acceleration of bone maturation; no change in eGFR; increase in IGF-I and integrated insulin levels during GH
Fine et al. (1995) [S17] (USA)	Multicenter, RCT, open labeled	n=30; age <2.5 yrs.<br><br>GH: n=19 (M/F16/3); CKD stage 3–5<br><br>Controls: n=11 (M/F 7/4); CKD 3–5	GH: 0.05 mg/kg/day<br><br>Controls: placebo	HV, 1st yr: 14.1 vs. 9.3 cm/y; 2nd yr: 8.6 vs. 6.9 cm/yr (each *P*<0.05). Delta height SDS, 2.0 vs. –0.2 during 2 yrs. (*P*<0.0001)
Hokken-Koelega et al. (1994) [S18] (Netherlands)	Multicenter, RCT, open labeled	n=23; CKD 4-5 n=8; CKD stage 5D n=15; prepubertal 23/23<br><br>GH 1; n=12 (M/F 11/1)	GH 1: 28 IU/m²/wk daily<br><br>GH 2: 14 IU/m²/wk daily	HV SDS comparable during 6 months; HV SDS higher at high dose in 2nd yr.; no further catch-up in 2nd year on low dose GH

		GH 2: n=11 (M/F 7/4)		
Hokken-Koelega et al. (1994) [S19] (Netherlands)	Multicenter, RCT, open labeled	n=16, KTx<br><br>GH 1: n=7 (M/F 4/3); prepubertal n=2, Tanner stage 2–3 n=5)<br><br>GH 2: n=9 (M/F 5/4); prepubertal n=4, Tanner stage 2–3 n=5	GH 1: 56 IU/m²/wk daily<br><br>GH 2: 28 IU/m²/wk daily	Height increment during 2 yr. GH treatment was 15.7 (5.1) cm and 5.8 (3.4) cm in controls (*P*<0.0001). Similar results in both GH groups
Fine et al. (1994) [S20] (USA)	Multicenter, RCT, double blinded	GH: n=82 (M/F 61/21); prepubertal 82/82<br><br>Control: n=43 (M/F 28/14) prepubertal 43/43<br><br>eGFR <75 ml/min/1.73 m^2	GH: 0.05 mg/kg/d<br><br>Control: placebo	Height SDS after 2 yrs:<br>GH –1.55 vs. –2.94;<br>Controls, –2.91 vs. –2.82 (*P*<0.0001).<br>HV: GH, 10.7 cm/yr (1st yr), 7.8 cm/yr (2nd yr);<br>controls, 6.5 cm/yr (1st yr), 5.5 cm/yr (2nd yr); each *P*<0.0001)
Hokken-Koelega et al. (1991) [S21] (Netherlands)	Multicenter, RCT, double blinded cross-over	GH: n=8 (M/F 6/2)<br><br>Controls: n=8 (M/F 4/4)<br><br>eGFR <20 ml/min/1.73 m^2	GH: 28 IU/m²/wk for 6 months<br><br>Controls: placebo	HV in GH group was significantly higher compared to controls by 2.9 cm per 6 months (*P*<0.05)

Data are given as mean ± SD if not indicated otherwise. CKD, chronic kidney disease; CT, conservative treatment (CKD prior to dialysis); eGFR, estimated glomerular filtration rate; F, female; GH, growth hormone; HV, height velocity; KTx, kidney transplantation; M, male; PD, peritoneal dialysis; RCT, randomized controlled trial; s.c., subcutaneous; SDS, standard deviation score; SE, standard error

Supplementary Table 3 | Uncontrolled trials and observational studies (n=33)

Ref. (country of origin)	Study design	N	Population characteristics	GH dosage	Outcomes
Nawrot-Wawrzynia et al. (2013) [S23] (Austria)	Observational study	18	CKD stage 5, n=18 (M/F 13/3) age 3.6–16 yrs. pubertal stage Tanner stage 1: n=15/18 Tanner stage 2: n=3/18	1.0–1.1 IU/kg/wk daily for 12 months	• Bone mineralization density distribution: patients had low bone turnover (*P*<0.05); heterogeneity in mineralization.<br>• After GH treatment, height increased by 9.1 cm (*P* <0.001) and bone turnover indices to normal values or beyond<br>• Lower and more heterogeneous matrix mineralization compared to baseline
Youssef et al. (2012) [S24] (Egypt)	Crossover non-randomized controlled clinical trial	15	CKD stage 5, n=15 (M/F 7/8) age 10.6 ± 2.8 yrs (range 5-14 yrs.); pubertal stage not given	0.33 mg/kg/wk (0.8 IU/kg/wk) three times per wk for 1 year	• The year before therapy, increase of height was not statistically significant (*P* >0.05)<br>• The year before therapy growth velocity was 0.6 cm/year<br>• Under GH therapy, height increase was statistcally not significant (*P*>0.05)<br>• Under GH therapy: growth velocity, 4.1 cm/year
Müller-Wiefel et al. (2010) [S25] (Germany)	Open-label, international, multicenter study	81	CKD stage 3–5 n=37; CKD 5D n=27; KTx n=17 (M/F 58/23); age 8.6 ± 3.9 yrs; pubertal stage not given	0.35 mg/kg/wk daily for 12 months then extended to 2–5 yrs.	Change in HV and height versus baseline<br>• After 12 months of treatment: HV: 4.6 ± 3.1 to 9.0 ± 3.6) cm/yr (*P*<0.001). Mean height SDS: –3.7 ± 1.7 to –3.0 ±1.7 (*P*<0.001). Mean HV SDS –2.4 ± 2.5 to 3.8 ± 4.5 (*P*< 0.001).<br>• After 24 months of treatment: HV: 4.5 ± 3.3 to 7.5 ± 2.9 cm/yr (*P*<0.001). Mean height SDS: -3.6 ± 1.5 to –2.5±1.5 (*P*<0.001). Mean HV SDS: -2.4 ± 2.2 to 1.1 ± 0.8 (*P*<0.001).<br><br>Normal height SDS was noted in 1% of children at baseline, 17% after 12 months and 43% after 24 months of GH therapy
Mencarelli et al. (2009) [S26] (Italy)	Retrospective study	27	CKD stage 3–5D Infants.<br><br>GH: n=12 (M/F 9/3 Controls: n=15 (M/F 11/4).<br><br>Higher frequency of ESRD in GH group	0.24 ± 0.07 mg/kg/wk daily	Height SDS: between the age of 0.5 and 2.5 years, the height SDS increased from –2.0 ± 1.2 to –0.9 ± 0.9 in the GH group (*P* < 0.005) and from –1.6 ± 1.6 to –1.0 ± 1.9 in the control group (*P*>0.05)
Kari et al.	Retrospective	32	CKD stage 3-5	28 IU/m2/wk	CKD stage 3–5: height SDS

(2005)<br>[S27]<br>(Saudi Arabia)	study		n=21; CKD 5D n=11 (M/F 23/9);<br>age: 8.3 ± 3.7 yrs; pubertal stage not given	daily until KTx over a mean period of 3.7 ± 2.0 years	improved from<br>–2.5±1.4 to –2.1±0.7 at 1 yr, –2.0±0.7 at 2 yrs, and<br>–1.6±0.6 at 3 yrs (each *P*<0.05).<br>CKD stage 5D: height SDS improved from –2.7±0.5 to –2.3±0.5 at 1 yr (*P*<0.05). Thereafter, no further change.
Gipson et al. (2001)<br>[S28]<br>(USA)	Prospective study	9	CKD stage 5D n=9; gender distribution and age not given; all prepubertal	0.05 mg/kg/d, intraperitoneal	Height SDS was<br>–3.1 at baseline, –2.5 at 1 yr, and –2.3 at 2 yrs (*P*>0.05).<br>Mean HV increased from 4.6 cm/yr to 8.5 cm/yr in the 1st yr (*P*<0.05) and 6.1 cm/yr during 2nd yr (*P*>0.05 vs. baseline). No increased peritonitis infection rates.
Hokken-Koelega et al. (2000)<br>[S29]<br>(Netherlands)	Multicenter, controlled, follow-up of previous trial: [S18,S21]	45	CKD stage 5D n=27 (PD:HD 18:9); CKD stage 3–5, n=18 (M/F 28/17); age 7.3 yrs; all prepubertal	3.8 IU/m2/d for a maximum of 8 yrs	Significant increment in mean height SDS over baseline values (*P*<0.001), both in the total group of children with intermediate-and long-term GH therapy (n=45) as well as in those treated with GH for 6 (n=11) and 8 yrs (n=7).
Haffner et al. (1998)<br>[S30]<br>(Germany)	Multicenter prospective study	103	CKD stage 3–5D n=74 (eGFR 26 ± 2 ml/min/1.73m²);<br>CKD stage 5D n=29 (M/F 70/33)<br>Age 8.5 yrs.<br>all prepubertal	28 to 30 IU/m^2/wk daily up to 5 yrs.	Height SDS in CKD stage 3–5:<br>Baseline –3.4 ± 0.1<br>1st yr –2.6 ± 0.1<br>2nd yr –2.1 ± 0.2<br>3rd yr –1.8 ± 0.3<br>4th yr –1.7 ± 1.5<br>5th yr –1.9 ± 1.5<br>(each *P*<0.05)<br>Height SDS in CKD stage 5D<br>Baseline –3.6 ± 0.2<br>1st yr –3.1 ± 0.3<br>2nd yr –3.0 ± 0.4<br>3rd yr –3.7 ± 0.8<br>(each *P*<0.05).<br>Predicted adult height (+7.7 cm) after 3 yrs of GH treatment (*P*< 0.001)
Bérard et al. (1998)<br>[S31]<br>(France)	Multicenter prospective study	42	CKD stage 5D n=42 (M/F 26/16),<br>age 10.4 ± 4.5 yrs.<br>34/42 prepubertal<br>8/42 early puberty	1 IU/kg/wk daily for 1-5 yrs	1st year of GH, HV increased from 3.5 to 7.0 cm/year (*P*<0.0001) and was always over 2.5 cm/yr.<br>Height SDS increased by 0.5 SDS.<br>-No significant adverse effects were observed
Wühl et al. (1998)<br>[S32]<br>(German)	Multicenter prospective study	36	Cystinosis patients; only<br>CKD stage 2–5D (eGFR 50 ± 27 ml/min/1.73m²)<br>(M/F 20/16),<br>age 7.3 ± 2.7 yrs; pubertal status not given	1 IU/kg/wk daily for upto 5 yrs	During the 1st year HV increased from 4.1 ± 1.6 cm/yr to 8.8 ± 2.5 cm/yr.<br><br>Height SDS improved within 1 yr from –4.2± 1.0 to –3.3 ± 1.0 (each *P*<0.05)
Wühl et al. (1996)<br>[S33]<br>(Germany)	Prospective study	56	CKD stage 3–5, eGFR 26 ± 17 ml/min/1.73m²<br>(M/F 26/12), n=38,	28–30 IU/m2 /wk for upto 2 yrs.	HV: CKD stage 3–5, 4.9 cm/yr to 9.5 cm/yr; CKD 5D: 4.6 cm/yr to 7.3 cm/yr (each *P*<0.05)

			age 6.5 ± 2.4 yrs.<br><br>CKD stage 5D, n=18 (M/F 6/12), age 6.5 ± 2.0 yrs. all prepubertal		Delta height SDS: CKD stage 3–5, 1.1 (1st yr), 0.5 (2nd yr); CKD stage 5D 0.5 (1st yr), 0.2 (2nd yr); each *P*<0.05
Lanes et al. (1996) [S34] (Venezuela)	Prospective study	13	CKD stage 3–5 (eGFR 21 ± 18 ml/min/1.73m²) (M/F 11/2) age 6.7 ± 3.4 yrs. (range 2.5 to 12.0 yrs), all prepubertal	1 IU/kg/wk daily for 12 months	HV increased from 4.3 cm/yr to 9.1 cm/yr and height SDS from –3.5 to 2.6 (each *P*<0.05).<br>GH treatment resulted in normalization of formally reduced bone mineral density.
Maxwell et al. (1996) [S35] (Canada)	Prospective study	8	GFR stage 3–5 (M/F 5/3), age 1.9 yrs. (1.3–2.7), all prepubertal	0.14 IU (0.05 mg)/kg/d	Height SDS improved from –3.3 to –2.2 (*P*<0.01).<br>HV SDS improved from –1.3 to 1.1 (*P*<0.01).<br>No Change in eGFR
Schwartz et al. (1995) [S36] (USA)	Prospective study	15	CKD stage 5D n=6 (M/F 4/2);<br>KTx n=9 (M/F 8/1); age 9.1 ± 2.5 yrs; pubertal stage not given	0–6 months, 0.16 ± 0.02 mg/kg/week daily; 6–12 months, 0.22 ± 0.7 mg/kg/week daily	HV SDS increased in both groups compared to baseline (each *P*<0.05);<br>no significant increase in height SDS
Fine et al. (1994) [S37] (USA)	Prospective study	11	CKD stage 2–5 (eGFR 5–75 ml/min/1.73m²) (M/F 11/0),<br>age 2.5–16.3 yrs; pubertal stge not given	8 patients: 0.125 mg/kg thrice weekly for 6 months, then 0.053 mg/kg daily for up to 60 months.<br>3 patients: 0.053 mg/kg daily for up to 60 months	HV increased from 5.4 to 8.9, 7.4 (*P* <0.05), 7.6 (*P*< 0.01), 6.5 (*P* <0.05), and 7.5 cm/yr (p=NS) for each year.<br>Height SDS increased from –3.2 to –0.85 at 60 months (*P*<0.001)
Schaefer et al. (1994) Germany [S38]	Prospective study	31	HD: n=14 (M/F 6/8); age 11.8 ± 3.7 yrs.<br><br>CPD n=17 (M/F 11/6)<br>age 8.7 ± 4.3 yrs. all prepubertal	28-30 IU/m2/week daily for 12 to 24 months	HV: HD, 5.5 vs. 2.9 cm/yr; PD, 7.2 vs. 3.0 cm/yr (each *P*<0.001)
Jabs et al. (1993) [S39] (USA)	Prospective study	8	KTx: age 7.4 to 17.7 yrs; prepubertal and pubertal pts	0.05 mg/kg/day for daily 12 months	HV increased from 1.7±0.7 to 7.1±2.1 cm/yr during the 1st yr. Height SDS increased from –3.9±1.5 to –3.4±1.3 (each *P*< 0.001)
Wühl et al. (1993) [S40] (Germany)	Prospective study	49	CKD stage 3–5D; KTx; all prepubertal	28 Prospective study 30 IU/m2/week daily for 12 months	Predictors of growth response to GH:<br>• HV inversely correlated with age (r = –0.63); and positively correlated with pretreatment HV (r = 0.65)<br>• Increment in HV SDS was negatively correlated with pretreatment HV SDS) (r = –0.58) each *P*<0.001<br>HV was highest in pts. on CT and lowest on dialysis

Tönshoff et al. (1993) [S41] (Germany)	Multicenter, prospective study	15	KTx (M/F 12/3), age 13.2 yrs; 10/15 pre-pubertal	30 IU/m2 per week daily for 36 months	HV in prepubertal pts (cm/yr): baseline 2.2<br>1st yr 7.9<br>2nd yr 7.2<br>3rd yr 5.5<br>(each *P*<0.05)
Van Renen et al. (1992) [S42] (Australia)	Prospective study	9	CKD stage 3–4; eGFR 11–60 ml/min/1.73m² (M/F 9/0), age 4.8–15.6 yrs; prepubertal 5/9	30 IU/m²/wk daily for 12 months	Prepubertal: HV increased from 4.6±1.3 to 9.0±1.3 cm/yr (*P*<0.001); height SDS increased from –2.2±0.7 to –1.5±0.5 (*P*<0.01).<br>Pubertal: HV increased from 5.4±1.4 to 10.4±1.8 cm/yr (*P*<0.01); height SDS increased from –1.9±0.7 to –1.3±0.9 (*P*<0.05).
Fine et al. (1992) [S43] (USA)	Prospective study	13	KTx (M/F 11/2), age 7.6 to 17.7 yrs; prepubertal and pubertal pts.	0.375 mg/kg per week given daily for 12-36 months	HV SDS increased from 2.7 to 6.3 (12 mo.) and to 5.2 (24 mo.); each *P*<0.05.<br>eGFR was 66±26 mL/min/1.73m² at baseline, 55±30 ml/min/1.73m² at 1 yr and 52±28 ml/min/1.73m2 at 2 yrs. (each P>0.05).
Van Dop et al. (1992) [S44] (USA)	Prospective study	9	KTx, age 12.6±4.0 yrs; 7/9 prepubertal	0.3 to 0.35 mg/kg/wk given daily, three times per wk, or six times per wk	HV: 1.9 ± 1.1 cm/yr to 7.2 ± 1.8 cm/yr (*P*<0.01)
Bartosh et al. (1992) [S45] (USA)	Prospective study	5	KTx (M/F 4/1), age 15.2±2.0 yrs; all prepubertal	0.05 mg/kg/wk, given 6 times/wk for 12 months	HV (cm/y): baseline 3.5, 1st yr 8.5 (*P*<0.05).<br>Height SDS: –4.3 vs. –4.9 (*P*<0.05)
Fine et al. (1991) [S46] (USA)	Prospective study	9	CKD stage 2–5 eGFR 5–75 ml/min/1.73m² (M/F 9/0), age 2.8-16.3 yrs; pubertal stage I–II	0.05 mg/kg/day for 1 to 3 yrs	HV increased significantly during GH compared to baseline.<br>No significant change in eGFR
Van Es et al. (1991) [S47] (Sweden)	Prospective study	74	CKD stage 3–5 n=31; prepubertal 31/31<br><br>KTx n=43; 26/43 prepubertal	28–30 IU/m2 per week daily for 24 months	HV (cm/y): CKD stage 3–5, 9.8 (6.8, 2nd yr) vs. 4.2.<br>Prepubertal KTx: 8.4 (5.4 2nd yr) vs. 3.6.<br>Pubertal KTx: 6.6 (4.5 2nd yr) vs. 3.2.<br>(each *P*<0.05)
Tönshoff et al. (1991) [S48] (Germany)	Prospective study	43	CKD stage 3–5 n=17; prepubertal 7/17<br><br>CKD stage 5D n=13; prepubertal, n=10<br><br>KTx n=13; prepubertal 10/13	28-30 IU/m2 per week daily for 12-24 months	Prepubertal pts. HV (cm/yr):<br><br>CKD 3–5 10.0 (9.3, 2nd yr) vs. 4.3 (each *P*<0.05).<br><br>CKD stage 5D 7.3 vs. 4.2.<br><br>KTx, 7.9 (8.6, 2nd yr) vs. 2.3 (each *P*<0.05)
Rees et al. (1990) [S49] (UK)	Prospective study	18	CKD stage 4–5 n=6; all prepubertal; age 7.7 yrs (5.0–10.4) (M/F 5/1)	30 IU/m2 per wk. daily for 12 months	HV:<br>CKD stage 4–5: 4.8 to 10.1 cm/yr<br>KTx (prepubertal): 2.3 to 6.1 cm/yr

			KTx n=6;majority prepubertal;age 12.1 yrs (9.5–15.8) (M/F 3/3)<br><br>KTx n=6; all pubertal;age 15.6 yrs (14.1–18.3) (M/F 4/2)		KTx (pubertal): 3.2 o 6.0 cm/yr (each *P*<0.05)
Tönshoff et al. (1990) [S50] (Germany)	Prospective study	9	CKD stage 5 n=1; CKD 5D n=8 (M/F 7/2); age 5.8 yrs; all prepubertal	4 IU/ m^2 per day for 12 months	HV (cm/y): 8.0 vs. 4.4<br>HV SDS increased from –2.6 to 1.5<br>(each *P*<0.05)
Johansson et al. (1990) [S51] (Belgium)	Prospective study	50	CKD stage 4–5 n=22, all prepubertal; age 8.4 yrs (3.1– 12.8)<br><br>KTx n=15, all prepubertal<br><br>KTx n=13, all pubertal	28-30 IU/m2 per wk. daily for 12 months	CKD stage 4-5:<br>HV increased from 4.8 cm/yr to 10.0 cm/yr (HV SDS from –1.3 to 5.1)<br><br>KTx prepubertal children:<br>HV increased from 2.6 cm/yr to 6.2 cm/yr (HV SDS from -2.8 to 2.3)<br><br>KTx pubertal children:<br>HV increased from 3.8 cm/yr to 6.7 cm/yr<br>(each *P*<0.05)
Fine et al. (1990) [S52] (USA)	Prospective study	5	CKD stage 5D (PD), age 1.2 to 17.7 yrs; prepubertal and pubertal patients	0.125 mg/kg 3 times weekly for 12 months	Significant increase in HV compared to pretreatment year (*P*<0.05)
Tönshoff et al. (1989) [S53] (Germany)	Prospective study	9	CKD stage 5D (M/F 7/2); all prepubertal	4 IU/ m2 per day for 6–9 months	HV SDS changed from –2.8 to 2.5 (*P*<0.05)
Koch et al. (1989) [S54] (USA)	Prospective study	5	CKD stage 3–5D (eGFR 18±6 ml/min/1.73 m²); (M/F 5/0);<br>age 4.6±1.8 yrs;<br>all prepubertal	0.125 mg/kg 3 times weekly for 12 months	HV (cm/yr) increased from 4.9±1.4 to 8.9±1.2, and height SDS from -3.0± 0.7 to –2.4±0.8 (each *P*<0.05)
Lippe et al. (1988) [S55] (USA)	Prospective study	5	CKD stage 3–5<br>age 35–91 months<br>all prepubertal	0.125 mg/kg 3 times weekly for 6 months and 6 months follow-up without GH	HV (cm/yr) increased from 4.9±1.4 to 10.1± 2.0 (*P*<0.01)

Data are given as mean ± SD if not indicated otherwise. CKD, chronic kidney disease; CPD, continuous peritoneal dialysis; CT, conservative treatment (CKD prior to dialysis); eGFR, estimated glomerular filtration rate; ESRD, end-stage renal disease; F, female; HD, haemodialysis; HV, height velocity; GH, growth hormone; KTx, kidney transplantation; M, male; NS, nonsignificant; SDS, standard deviation score; SE, standard error.

Suppl. Table 4: Synopsis of 11 studies reporting adult height or near adult height data after GH treatment of growth failure in CKD patients.

1st author, year, origin [Ref.]	Study design	Patients	Age at start of GH (years)	Pre-pubertal (%)	Duration of follow-up (years)	GH dosage[a]	Duration of GH Tx (years)	Initial height SDS	Adult height SDS[b]	Change in height SDS
Gils S 2018, Argentina[c] [S56]	Prospective study	KTx; n=23 (only boys)<br>GH, n=13<br>no GH, n=10	15.5	0	3.1	9.33 mg/m^2/wk daily	2.3	-3.1 ± 1<br>-2.5 ± 1.1<br>(p>0.05)	-1.8 ±0.8<br>-2.9 ±1<br>(p<0.05)	1.2 ± 0.3<br>(p<0.01)<br>-0.3 ± 0.3<br>(p>0.05)
Gils S 2012, Argentina [S57]	Prospective study	KTx, n=47<br>GH, n=33,<br>no GH, n=14	13.2<br>12.3	45<br>57	≥ 3	10 mg/m^2/wk daily	3.5	-3.3 ± 1.2<br>-3.0 ± 0.7<br>(p>0.05)	-1.9 ± 1.1<br>-3.5 ± 1.2<br>(p<0.05)	1.2 ± 0.7,<br>(p<0.01)<br>-0.5 ± 0.2,<br>(p<0.05)
		CKD stage 3-5, n=35	n.i.		n.i.		n.i.	-3.0 ± 0.9	-1.8 ± 0.9	1.2
Berard E 2008, France [S58]	Prospective study	HD, n=19	n.i.	63	n.i.	1 IU/kg/wk daily	n.i.	-4.1 ± 0.9	-2.5 ± 1.2	1.6
		KTx, n=48	n.i.		n.i.		n.i.	-3.2 ± 11<br>(p>0.05)	-2.2 ± 1.2<br>(each p<0.05[d])	1.0<br>(each p<0.05)
Nissel R 2008, Germany [S59]	Registry	CKD 3-5, n=108<br>dialysis, n=67<br>KTx, n=65<br>n=240<br>regular pubertal onset<br>delayed puberty<br>in early puberty<br>in late puberty	12.8	n.i.	4.9	0.33 mg/kg/wk daily	> 2	-3.2	-1.7	1.5
			14.2	n.i.	4.0			-4.0	-3.0	1.1
			13.7	n.i.	4.1			-3.6	-2.4<br>(each p<0.05[d])	1.1<br>(each p<0.002)
			10.0	100	n.i.			-3.3	-2.0	1.3
			14.2	100	n.i.			-4.9	-3.6	1.3
			14.6	0	n.i.			-3.1	-2.2	0.9
			16.4	0	n.i.			-3.9	-2.9<br>(each p<0.05[d])	1.0<br>(each p<0.05)
Seikaly MG 2007, USA	Registry	n=91								

[S60]		CKD 3-5, n=30	n.i.	ca. 60	n.i.	n.i.	> 2	-2.6	n.i.	0.8
		dialysis, n=20	n.i.	ca. 60	n.i.	n.i.	> 2	-2.7	n.i.	0.5
		KTx, n=41	n.i.	ca. 60	n.i.	n.i.	> 2	-2.4	n.i.	0.19<br>(each<br>p<0.05)
Fine RN 2005,<br>USA<br>[S61]	Registry	KTx, n=676<br>GH, n=71<br>non-GH, n=669	n.i.	ca. 50	n.i.	n.i.	> 2	-2.7<br>-2.5<br>(p>0.05)	-1.8<br>-2.6<br>(p<0.001)	0.9 (p<0.001)<br>-0.1 (p>0.05)
Crompton C<br>2004<br>Australia<br>[S62]	Registry	n=39<br>CT, dialysis, KTx	12.8	ca. 50	5.4	27 IU/m^2/wk<br>daily	3.3	-2.65	-2.3	0.35<br>(p<0.001)
Hokken-<br>Koelega AC<br>2004, The<br>Netherlands[e]<br>[S63]	Prospective<br>study	n=65<br>CT, dialysis	n.i.<br>15.5	100<br>0	n.i.<br>n.i.	4 IU/m^2/d	5.8<br>n.i.	-2.8<br>n.i.	-1.4<br>n.i.	1.4 (p<0.001)<br>height gain<br>19 cm
Fine RN 2000,<br>USA<br>[S64]	Registry	CT, GH, n=9<br>CT, non-GH, n=335	n.i.	n.i.	3.2	n.i.	< 3.2	-3.0<br>-1.0	-2.3<br>-1.0	0.7 (p<0.05)<br>-0.02<br>(p<0.05)
		dialysis, GH, n=22<br>dialysis, non-GH, n=377	n.i.	n.i.	4.1	n.i.	< 4.1	-3.6<br>-1.88	-3.2<br>-1.82	0.4 (p=0.09)<br>0.06 (p=0.09)
		KTx, GH, n=72<br>KTx, non-GH, 1480	n.i.	n.i.	3.7	n.i.	< 3.7	-3.0<br>-1.7	-2.5<br>-1.7	0.5 (p<0.01)<br>0.04 (p<0.01)
Haffner D<br>2000, Germany<br>[S65]	Prospective<br>study	GH, n=38<br>47% CKD 3-5, 24%<br>dialysis, 29% KTx[f]<br>non-GH, n=50<br>53% CKD 3-5, 20%<br>dialysis, 27% KTx[f]	10.4<br>9.7	100<br>100	7.6<br>8.3	0.33 mg/kg /wk<br>daily	5.3<br>-	-3.1<br>-1.5<br>(p<0.05)	-1.6 ± 1.2<br>-2.1 ± 1.2<br>(p<0.05)	1.4 (p<0.001)<br>-0.6 p<0.001)
Janssen F<br>1997, Belgium<br>[S66]	Retrospective<br>study	KTx, n=17	n.i.	n.i.	n.i.	4 IU/m^2/d	3.4	-3.0	-1.8	1.2 (p<0.05)

[a]1 IU = 0.33 mg; [b]follow up / subanalysis of Gils S, 2012; [c]in the studies of Gils et al [S56] and Nissel et al [S59] near adult height data were reported; [d]vs. baseline; [e]published only in abstract form; [f]percentage distribution of patient years spent in each treatment category; Tx = treatment; CT = conservative treatment (CKD prior to dialysis); KTx = Kidney transplantation;
n.i. = no information given

Supplementary Table 5 | Model parameters, values and data sources for cost-effectiveness of GH in CKD.

Scenario	Parameter	Value and source	Mean total cost of GH therapy	Incremental cost per cm gained
**Population data**				
All scenarios	Sex distribution of patients	50% males	NA	NA
**Investigation and treatment parameters**				
All scenarios	Drug doses condition based on age- and sex-related weight at 25th percentile and not adjusted during puberty.	0.045 mg/kg per day	NA	NA
All scenarios	Median cost per mg	€22	NA	NA
**Effectiveness data**				
Scenario 1A[a]	Length of treatment	2 years	€12,966	€1,805
	Final height gain	7.2 cm		
Scenario 1B[a]	Length of treatment	5 years	€37,905	€5,265
	Final height gain	7.2 cm		
Scenario 2A[b]	Length of treatment	2 years	€ 27,075	€ 3,760
	Final height gain	7.2 cm		
Scenario 2B[b]	Length of treatment	5 years	€80,142	€11,131
	Final height gain	7.2 cm		

[a]Assumes a child aged 5 years and benefit uniformly sperad over treatment period. [b]Assumes a child aged 12 years and benefit uniformly sperad over treatment period. NA, not applicable.

Supplementary Table 6 | Costs of GH in eight representative European countries in 2018

Country	Median cost for 1 mg of GH reference (somatotropin)	Median cost for 1 mg of GH biosimilar	Median cost for 1 mg GH
Belgium	€23	€20	€22
France	€30	€25	€28
Germany	€60	€48	€54
Italy	€29	€15	€22
Netherlands	€30	€30	€30
Poland	€10	€4	€7
Spain	€16	n.a.	€16
United Kingdom	€22	€17	€20
Median			**€22**

Costs were obtained from national data sources or local pharmacies; n.a., not available.

Supplementary Table 7 | Adverse events in parallel RCTs comparing GH versus control group

Reported adverse effects	Studies [Ref.]	N (GH, control)	Control group	GH group	Between groups comparison	rhGH discontinuation
Benign intracranial hypertension (ICH)	Fine 2002 (KTx) [S10]	68 (29, 39)	At 1st year: 1 patient	At 1st year: 1 report of headache with normal cerebrospinal fluid pressure.	-	Both patients discontinued from study.
	Broyer 1998 (KTx) [S20]	90 (46, 44)	1 patient developed papilledema while on GH – group not specified		-	Papilledema resolved after discontinuation of GH.
Bone histology changes	Sanchez 2002 (KTx) [S15]	23 (11, 12)	At 1st year, 1 patient developed mild lesion of secondary hyperparathyroidism on bone biopsy (n=8)	At 1st year, 2 patients developed adynamic bone and 2 patients developed mild secondary hyperparathyroidism (N=8)	-	None reported
Glucose intolerance	Fine 2002 (KTx) [S7]	68 (29, 39)	At 1st year (no treatment): 0 report<br>At 2nd year (GH): 1 patient developed hyperglycaemia	At 1st year: 1 patient developed diabetes mellitus.	-	GH discontinued in the patient with diabetes mellitus; reintroduction of GH with no problem.
	Broyer 1998 (KTx) [S14]	90 (46, 44)	At year 1: increase in mean fasting glucose concentrations, fasting plasma insulin, mean values of insulin during OGTT. (N=19)<br>1 children developed diabetes during 1st year (before GH)	At year 1: increase in mean fasting glucose concentrations, fasting plasma insulin, mean values of insulin during OGTT. (n=20)	NS	None reported
	Maxwell 1998 (KTx) [S10]	22 (9, 13)	No report	At 9 months of GH therapy, 1 patient with partial pancreatectomy had raised fasting glucose, insulin, and HbA1c concentrations.		GH was discontinued and values returned to normal.

Supplementary Table 7 – continued

Reported adverse effects	Studies	N (GH, control)	Control group	rhGH group	Between groups comparison	rhGH discontinuation
Graft rejection	Broyer 1998 (KTx) [S14]	90 (46, 44)	Acute, biopsy-proven rejection:<br>1st year (no treatment): 4 patients<br>2nd year (GH): 6 patients	Acute, biopsy-proven rejection:<br>1st year: 9 patients<br>2nd + 3rd year: 12 patients[a]	1st year: NS	4 pts. discontinued GH, recovered and maintain stable renal function. A total of 13 cases of discontinuation.[a]
	Fine 2002 (KTx) [S7]	68 (29, 39)	Rejection episodes:<br>At 1st year (no treatment): 3 patients<br>At 2nd year (GH): 2 patients<br>Allograft failure: 1 patient at 2nd year while on GH	Rejection episodes:<br>At 1st year: 0 report<br>At 2nd year: 3 patients<br><br>Allograft failure: 2 patients	-	None reported
	Sanchez 2002 (KTx) [S8]	23 (11, 12)	No report	2 patients had biopsy confirmed acute rejection after 3 and 12 months of GH therapy.	-	None reported
	Maxwell 1998 (KTx) [S10]	22 (9, 13)	Presumed rejection episodes:<br>At 1st year: 9 patients	Presumed rejection episodes:<br>At 1st year: 8 patients	NS	None reported
Renal function deterioration	Fine 2002 (KTx) [S7]	68 (29, 39)	At 1st year (no treatment): 0 report<br>At 2nd year (GH): 2 patients with elevated serum creatinine	None reported	-	None reported
	Broyer 1998 (KTx) [S14]	90 (46, 44)	At 1st year: Moderate but significant decrease in GFR.	At 1st year: Moderate but significant decrease in eGFR	NS	7 cases discontinued due to increased serum creatinine level[a]
	Fine 1994 (CT) [S20]	125 (43, 82)	At 2nd year: Serum creatinine levels rose (n=24)	At 2nd year: Serum creatinine levels rose (n=48)	NS	None reported

Supplementary Table 7 – continued

Reported adverse effects	Studies	N (GH, control)	Control group	rhGH group	Between groups comparison	rhGH discontinuation
Others	Sanots 2010 (CT/CKD VD) [S5]	14 (7, 7)	20 unspecified adverse events	9 unspecified adverse events. None were considered related to rhGH therapy.	*P*=0.065	None reported
	Fine 2002 (KTx) [S7]	68 (29, 39)	At 1st year (no treatment):<br>2 cases of infection; 1 case of septic arthritis; 1 patient developed post-transplant lymphoproliferative disease<br><br>At 2nd year (GH):<br>1 case of seizure; 1 case of esophogeal bleeding	At 1st year: 2 cases of infection; 1 case of transient ischemia attack; 1 case of genu valgum; 1 patient developed post-transplant lymphoproliferative disease.<br><br>1 patient developed Hodgkin's disease at 36 months	-	None reported
	Maxwell 1998 (KTx) [S10]	22 (9, 13)	No report	1 patient developed worsening of a pre-exisintg idiopathic scoliosis.	-	None reported
	Fine 1994 (CT) [S20]	125 (43, 82)	At 2nd year: 0 report of asthma/ wheezing (n=27)	At 2nd year: 8 reports of asthma/ wheezing (n=55)	*P*=0.048	None reported
Reported "no adverse effects"	Bacchetta 2013 (CKD VD) [S4]<br>Hokken-K 1991 (CT/CKD VD) [S21]<br>Hokken-K 1996 (KTx) [S16]					
Adverse effects not addressed	Powell 1997 (CT) [S11]Kuizo 1998 (CKD VD) [S9]					

KTx, Kidney transplant; CT, conservative treatment (CKD prior to dialysis); CKD 5D, dialysis; N, total no. of patients randomized (no treatment group, GH group); NS, Non-significant; OGTT, Oral glucose tolerance test.

Supplementary Table 8 | Adverse events in parallel RCTs comparing two doses of GH

Reported adverse effects	Studies	N (low dose, high dose)	GH group (2 IU/m^2/day)	GH group (4 IU/m^2/day)	GH discontinuation
Claudication	Kitagawa 1997 (CT/CKD VD) [S13]	122 (54, 58)	Number of cases not specified		None reported
Graft rejection	Ito 1997 (KTx) [S12]	23 (10, 13)	Acute, biopsy confirmed rejection<br>At 1st year: 2 patients	Acute, biopsy confirmed rejection<br>At 1st year: 5 patients	None reported
Glucose intolerance	Hertel 2002 (CT/CKD VD) [S6]	29 (15, 14)	1 patient developed diabetes mellitus after 34 months of therapy.<br>At 2nd year (4 IU/m^2/day): significant increase in fasting insulin levels	0 reports<br><br>At 2nd year (4 IU/m^2/day): significant increase in fasting insulin levels	Patient with diabetes mellitus discontinued GH
	Kitagawa 1997 (CT/CKD VD) [S13]	102 (54, 58)	2 cases reported[a]		None reported
Granuloma formation	Kitagawa 1997 (CT/CKD VD) [S13]	102 (54, 58)	2 cases reported[a]		None reported
Hypertension	Hertel 2002 (CT/CKD VD) [S6]	29 (15, 14)	1 patient after 6 months of therapy	0 reports	Hypertensive patient discontinued GH
	Kitagawa 1997 (CT/CKD VD) [S13]	102 (54, 58)	Number of cases not specified		None reported

Supplementary Table 8 - continued

Reported adverse effects	Studies	N (low dose, high dose)	GH group (2 IU/m²/day)	GH group (4 IU/m²/day)	GH discontinuation
Injection pain	Hertel 2002 (CT/CKD VD) [S16	29 (15, 14)	1 patient	1 patient	None reported
Lymph node swelling	Kitagawa 1997 (CT/CKD VD) [S13]	102 (54, 58)	Number of cases not specified		None reported
Renal function deterioration	Hertel 2002 (CT/CKD VD) [S6]	29 (15, 14)	At 1st year: 1 patient	At 1st year: 0 reports	None reported
	Kitagawa 1997 (CT/CKD VD) [S13]	102 (54, 58)	More patients in the 4 IU/m²/day than in the 2 IU/m²/day group showed signs of deterioration in renal function		None reported
	Callis 1996 (CT) [S67]	43 (21, 23)	At 6 months: 9 patients	At 6 months: 11 patients	None reported
Reported "no adverse effects"	Hokken-K 1994 (CT/CKD VD) [S18]				

N, Total no. of patients randomised (low dose, high dose); [a]It is not possible to determine whether these patients were from the low or high dose GH group.

Supplementary Table 9 | Summary of recommendations

	Recommendation	evidence quality, strength of recommendation
1.1	We recommend that height (or supine length for patients below 2 years of age) is regularly measured depending on age and chronic kidney disease (CKD) stage (Table 1). Height velocity should be calculated over a minimum period of 6 months, and both height and height velocity should be compared to standardized growth charts	A, strong
1.2	We recommend that growth potential is assessed by calculation of genetic target height on the basis of parental height and the extent to which the epiphysis of the left wrist is open on radiography (grade A, strong recommendation).<br><br>We do not recommend application of adult height prediction methods for children with CKD	A, strong<br><br>C, weak
1.3	Age, primary renal disease, systemic disorders, stage of CKD, dialysis adequacy (for patients on dialysis) and graft function and glucocorticoid therapy (in children post-transplantation) should be taken into account when considering growth hormone (GH) therapy.	B, moderate
1.4	CKD-associated growth-limiting factors such as protein-calorie malnutrition, metabolic acidosis, electrolyte disturbances (hyponatremia), dehydration and mineral dysregulation, including secondary hyperparathyroidism, should be adequately controlled before considering GH therapy (grade A, strong recommendation).	A, strong
1.5	The following assessments should be performed prior to starting GH:<br>• Serum creatinine (and estimated glomerular filtration rate), urea, calcium, phosphorus, total alkaline phosphatase, bicarbonate, parathyroid hormone, 25(OH) vitamin D, albumin, fasting glucose and glycosylated hemoglobin levels<br>• Serum thyroid hormone (TSH and free T3) and insulin-like growth factor 1 concentrations<br>• Fundoscopic examination<br>• Radiography of the left wrist<br>• Pubertal status according to Tanner	C, moderate
2.1	We recommend that pros and cons of growth hormone (GH) treatment are discussed with individual patients and their families before GH treatment is initiated. Such discussion is of particular importance for immobilized patients and those with syndromic kidney diseases.	no grading
2.2	We recommend that children with stage 3-5 chronic kidney disease (CKD) or on dialysis aged above 6 months should be candidates for GH therapy if they have persistent growth failure, defined as a height below the third percentile for age and sex and a height velocity below the twenty-fifth percentile, once other potentially treatable risk factors for growth failure have been adequately addressed and provided the child has growth potential.	B, moderate
2.3	We recommend that GH therapy is considered for children with stage 3-5 CKD or on dialysis aged above 6 months who present with a height between the third and tenth percentile but persistent low height velocity (below the twenty-fifth percentile) once other potentially treatable risk factors for growth failure have been adequately addressed.	D, weak
2.4	In children who have received a kidney transplant and have persistent growth failure, defined as a height below the third percentile for age and sex and a height velocity below the twenty-fifth percentile, we recommend initiating GH therapy 1 year after transplantation if spontaneous catch-up growth does not occur and steroid-free immunosuppression is not a feasible option.	B, moderate
2.5	In children with CKD due to nephropathic cystinosis who have persistent growth failure, defined as a height below the third percentile for age and sex and a height velocity below the twenty-fifth percentile, we recommend that GH therapy is considered at all stages of CKD.	C, moderate
2.6	GH therapy should not be started<br>• In patients with closed epiphyses<br>• In patients with known hypersensitivity to the active substance or to any of the excipients<br>• In the case of unwillingness of the patient or their family<br>• In patients with severe secondary hyperparathyroidism (parathyroid hormone > 500 pg/ml)	 X, strong<br>X, strong<br><br>X, strong<br>X, moderate<br>X, moderate

	• In patients with proliferative or severe non-proliferative diabetic retinopathy<br>• During the first year after renal transplantation<br>• In patients with acute critical illness<br>• In patients with active malignancy	X, moderate<br>X, strong<br>X, strong
3.1	We suggest considering the cost–benefit ratio before initiating growth hormone treatment in short children with chronic kidney disase.	D, weak
4.1	We recommend that growth hormone (GH) is given at a dose of 0.045–0.05 mg/kg body weight per day by subcutaneous injections in the evening.	B, moderate
4.2	We suggest that parents and physicians encourage children from about 8–10 years of age to do the GH injections on their own if adequate training and adherence is ensured.	D, weak
4.3	We recommend both GH reference and GH biosimilar products for use in short children with chronic kidney disease (CKD).	B, moderate
4.4	We suggest clinic visits every 3–6 months or more frequently for young patients and those with advanced CKD to monitor stature, height velocity, pubertal development, skeletal maturation on wrist radiography, renal function, thyroid hormone levels (TSH and free T3), serum glucose, calcium, phosphate, bicarbonate and parathyroid hormone levels.	D, weak
4.5	If height velocity in the first year of GH treatment is less than 2 cm per year over baseline, we recommend assessment of patient adherence to GH therapy, including measurement of serum insulin-like growth factor 1 levels, weight-adjusted GH dosage and assessment of nutritional and metabolic factors, as recommended before initiation of GH therapy.	B, moderate
4.6	We recommend stopping GH<br>• When epiphyseal closure is demonstrated<br>• At the time of renal transplantation<br>• In patients with persistent severe secondary hyperparathyroidism (parathyroid hormone (PTH) >500 pg/ml). GH may be reinstituted when levels return to the desired PTH target range<br>• With occurrence of intracranial hypertension<br>• In patients with slipped capital femoral epiphysis<br>• If the patient does not adequately respond to GH treatment despite optimal nutritional and metabolic control<br>• In patients with accelerated bone maturation<br>• In case of an unexplained decrease in estimated glomerular filtration rate	X, strong<br>X, strong<br>X, moderate<br>X, strong<br>X, strong<br>X, moderate<br>X, moderate<br>X, moderate
4.7	We suggest that cessation of GH treatment is considered<br>• When the patient reaches his or her genetic target height percentile. GH may be reinstituted if catch-down growth occurs<br>• When the patient reaches his or her genetic target height	X, moderate<br>X, moderate

References:

S 1 de Onis M, Onyango AW, Borghi E et al. Development of a WHO growth reference for school-aged children and adolescents. *Bull World Health Organ*. 2007; 85: 660-667.

S 2 Bryant J, Loveman E, Cave C, Chase D, Milne R. Endocrinology trial design: adverse event reporting in randomised controlled trials of recombinant human GH in GH-deficient adults. *J Endocrinol*. 2002175: 545-552.

S 3 Bonthuis M, van Stralen KJ, Verrina E, et al. Use of national and international growth charts for studying height in European children: development of up-to-date European height-for-age charts. *PLoS One*. 2012;7:e42506.

S 4 Bacchetta J, Wesseling-Perry K, Kuizon B, et al. The skeletal consequences of growth hormone therapy in dialyzed children: A randomized trial. *Clin J Am Soc Nephrol*. 2013; 8: 824-832.

S 5 Santos F, Moreno ML, Neto A, et al.. Improvement in growth after 1 year of growth hormone therapy in well-nourished infants with growth retardation secondary to chronic renal failure: results of a multicenter, controlled, randomized, open clinical trial. *Clin J Am Soc Nephrol* 2010; 5: 1190-1197.

S 6 Hertel NT, Holmberg C, Rönnholm KAR, et al. Recombinant human growth hormone treatment, using two dose regimens in children with chronic renal failure--a report on linear growth and adverse effects. *J Pediatr Endocrinol Metab*. 2002;15: 577-588.

S 7 Fine RN, Stablein D, Cohen AH, Tejani A, Kohaut E. Recombinant human growth hormone post-renal transplantation in children: a randomized controlled study of the NAPRTCS. *Kidney Int*. 2002; 62: 688-696.

S 8 Sanchez CP, Kuizon BD, Goodman WG, et al. Growth hormone and the skeleton in pediatric renal allograft recipients. *Pediatr Nephrol*. 2002; 17: 322–328.

S 9 Kuizon BD, Goodman WG, Gales B, Juppner H, Salusky IB. Effects of growth hormone on bone and mineral metabolism in dialyzed children [abstract]. *J Am Soc Nephrol;* 1998; 9: 546–547.

S 10 Maxwell H, Rees L. Randomised controlled trial of recombinant human growth hormone in prepubertal and pubertal renal transplant recipients. Arch Dis Child. 1998; 79: 481-487.

S 11 Powell DR, Liu F, Baker BK, et al. Modulation of growth factors by growth hormone in children with chronic renal failure. The Southwest Pediatric Nephrology Study Group. *Kidney Int*. 1997; 51: 1970–1979.

S 12 Ito K, Kawaguchi H. The use of recombinant human GH (rhGH) in growth failure in children with various renal diseases. *Clinical Pediatric Endocrinology* 1997; 6: 49–53.

S 13 Kitagawa T, Ito K, Ito H, Sakai T, Wada H, Kajiwara N. GH treatment of children with

chronic renal insufficiency: A Japanese clinical trial. *Clinical Pediatric Endocrinology* 1997; 6:73–80.

S 14 Broyer M. Results and side-effects of treating children with growth hormone after kidney transplantation – a preliminary report. Pharmacia & Upjohn Study Group. *Acta Paediatr Suppl*. 1996; 417: 76-79.

S 15 Kawaguchi H, Ito K. rhGH use in children with CRI and undergoing dialysis post-transplant in Japan: a multicentre study. MultiCenter Study Group Japan. *Br J Clin Pract Suppl.* 1996; 85: 26-31.

S 16 Hokken-Koelega AC, Stijnen T, de Jong RC, et al.. A placebo-controlled, double-blind trial of growth hormone treatment in prepubertal children after renal transplant. *Kidney Int Suppl*. 1996; 53: 128-134.

S 17 Fine RN, Attie KM, Kuntze J, Brown DF, Kohaut EC. Recombinant human growth hormone in infants and young children with chronic renal insufficiency. Genentech Collaborative Study Group*. Pediatr Nephrol.* 1995; 9: 451-457.

S 18 Hokken-Koelega AC, Stijnen T, de Jong MC, et al. Double blind trial comparing the effects of two doses of growth hormone in prepubertal patients with chronic renal insufficiency. *J Clin Endocrinol Metab*. 1994; 79: 1185-1190.

S 19 Hokken-Koelega AC, Stijnen T, de Ridder MA, et al. Growth hormone treatment in growth-retarded adolescents after renal transplant. *Lancet*. 1994; 343: 1313-1317.

S 20 Fine RN, Kohaut EC, Brown D, Perlman AJ. Growth after recombinant human growth hormone treatment in children with chronic renal failure: report of a multicenter randomized double-blind placebo-controlled study. Genentech Cooperative Study Group. *J Pediatr.* 1994; 124: 374-382.

S 21 Hokken-Koelega AC, Stijnen T, de Muinck Keizer-Schrama SM, et al.. Placebo-controlled, double-blind, cross-over trial of growth hormone treatment in prepubertal children with chronic renal failure. *Lancet* 1991; 338: 585–590.

S 22 Hodson EM, Willis NS, Craig JC. Growth hormone for children with chronic kidney disease. *Cochrane Database* Syst Rev. 2012; 2: CD003264

S 23 Nawrot-Wawrzyniak K, Misof BM, Roschger P, et al.. Changes in bone matrix mineralization after growth hormone treatment in children and adolescents with chronic kidney failure treated by dialysis: a paired biopsy study. *Am J* Kidney *Dis.* 2013; 61: 767-777.

S 24 Youssef DM. Results of recombinant growth hormone treatment in children with end-stage renal disease on regular hemodialysis. *Saudi J* Kidney *Dis Transpl*. 2012; 23: 755-764.

S 25 Müller-Wiefel D, Frisch H, Tulassay T, Bell L, Zadik Z. Treatment of growth failure with growth hormone in children with chronic kidney disease: an open-label long-term study. *Clin Nephrol*. 2010; 74: 97-105.

S 26 Mencarelli F, Kiepe D, Leozappa G, Stringini G, Cappa M, Emma F. Growth hormone treatment started in the first year of life in infants with chronic renal failure. *Pediatr Nephrol*. 2009; 24: 1039-1046.

S 27 Kari JA, Rees L. Growth hormone for children with chronic renal failure and on dialysis. *Pediatr Nephrol*. 2005; 20: 618-621.

S 28 Gipson DS, Kausz AT, Striegel JE, Melvin TR, Astrom LJ, Watkins SL. Intraperitoneal administration of recombinant human growth hormone in children with end-stage renal disease. *Pediatr Nephrol.* 2001; 16: 29-34.

S 29 Hokken-Koelega A, Mulder P, De Jong R, Lilien M, Donckerwolcke R, Groothof J. Long-term effects of growth hormone treatment on growth and puberty in patients with chronic renal insufficiency*. Pediatr Nephrol*. 2000; 14: 701-706.

S 30 Haffner D, Wühl E, Schaefer F, Nissel R, Tonshoff B, Mehls O. Factors predictive of the short- and long-term efficacy of growth hormone treatment in prepubertal children with chronic renal failure. the german study group for growth hormone treatment in chronic renal failure. *J Am Soc Nephrol*. 1998; 9: 1899-1907.

S 31 Berard E, Crosnier H, Six-Beneton A, Chevallier T, Cochat P, Broyer M. Recombinant human growth hormone treatment of children on hemodialysis. French Society of Pediatric Nephrology. *Pediatr Nephrol*. 1998; 12: 304-310.

S 32 Wühl E, Haffner D, Gretz N, et al.. Treatment with recombinant human growth hormone in short children with nephropathic cystinosis: no evidence for increased deterioration rate of renal function. The European Study Group on Growth Hormone Treatment in Short Children with Nephropathic Cystinosis. *Pediatr Res*. 1998; 43: 484-488.

S 33 Wühl E, Haffner D, Nissel R, Schaefer F, Mehls O. Short dialyzed children respond less to growth hormone than patients prior to dialysis. German study group for growth hormone treatment in chronic renal failure. *Pediatr Nephrol*. 1996; 10: 294-298.

S 34 Lanes R, Gunczler P, Orta N, et al.. Changes in bone mineral density, growth velocity and renal function of prepubertal uremic children during growth hormone treatment. *Horm Res*. 1996; 46: 263-268.

S 35 Maxwell H, Rees L. Recombinant human growth hormone treatment in infants with chronic renal failure. *Arch Dis Child*. 1996; 74: 40-43.

S 36 Schwartz ID[1], Warady BA, Buchanan CL, et al.. "Low-dose" growth hormone therapy during peritoneal dialysis or following renal transplantation. *Pediatr Nephrol.* 1995; 9: 320-324.

S 37 Fine RN, Yadin O, Moulton L, Nelson PA, Boechat MI, Lippe BM. Five years experience with recombinant human growth hormone treatment of children with chronic renal failure. *J Pediatr Endocrinol.* 1994; 7: 1-12.

S 38 Schaefer F, Wühl E, Haffner D, Mehls O. Stimulation of growth by recombinant human growth hormone in children undergoing peritoneal or hemodialysis treatment. German Study Group for Growth Hormone Treatment in Chronic Renal Failure. *Adv Perit Dial*. 1994; 10: 321-326.

S 39 Jabs K, Van Dop C, Harmon WE. Growth hormone treatment of growth failure among children with renal transplants. *Kidney Int Suppl*. 1993; 43: 71-75.

S 40 Wühl E, Haffner D, Tönshoff B, Mehls O. Predictors of growth response to rhGH in short children before and after renal transplantation. German Study Group for Growth Hormone Treatment in Chronic Renal Failure. *Kidney Int Suppl*. 1993; 43: 76-82.

S 41 Tönshoff B, Haffner D, Mehls O, et al.. Efficacy and safety of growth hormone treatment in short children with renal allografts: three year experience. Members of the German Study Group for Growth Hormone Treatment in Children with Renal Allografts. *Kidney Int.* 1993; 44: 199-207.

S 42 van Renen MJ, Hogg RJ, Sweeney AL, Henning PH, Penfold JL, Jureidini KF. Accelerated growth in short children with chronic renal failure treated with both strict dietary therapy and recombinant growth hormone. *Pediatr Nephrol.* 1992; 6: 451-458.

S 43 Fine RN, Yadin O, Moulten L, Nelson PA, Boechat MI, Lippe BH. Extended recombinant human growth hormone treatment after renal transplantation in children*.* *J Am Soc Nephrol*. 1992; 2: 274-283.

S 44 Van Dop C, Jabs KL, Donohoue PA, Bock GH, Fivush BA, Harmon WE. Accelerated growth rates in children treated with growth hormone after renal transplantation. *J Pediatr.* 1992; 120: 244-250.

S 45 Bartosh S, Kaiser B, Rezvani I, at al.. Effects of growth hormone administration in pediatric renal allograft recipients. *Pediatr Nephrol.* 1992; 6: 68-73.

S 46 Fine RN, Pyke-Grimm K, Nelson PA, et al.. Recombinant human growth hormone treatment of children with chronic renal failure: long-term (1- to 3-year) outcome. *Pediatr Nephrol*. 1991; 5: 477-481.

S 47 Van Es A. Growth hormone treatment in short children with chronic renal failure and after renal transplantation: combined data from European clinical trials. The European Study Group. *Acta Paediatr Scand Suppl*. 1991; 379: 42-48; discussion 49.

S 48 Tönshoff B, Dietz M, Haffner D, Tönshoff C, Stöver B, Mehls O. Effects of two years of growth hormone treatment in short children with renal disease. The German Study Group for Growth Hormone Treatment in Chronic Renal Failure. *Acta Paediatr Scand Suppl*. 1991; 379: 33-41.

S 49 Rees L, Rigden SP, Ward G, Preece MA. Treatment of short stature in renal disease with recombinant human growth hormone. *Arch Dis Child.* 1990; 65: 856-860.

S 50 Tönshoff B, Mehls O, Heinrich U, Blum WF, Ranke MB, Schauer A. Growth-stimulating effects of recombinant human growth hormone in children with end-stage renal disease. *J Pediatr*. 1990; 116: 561-566.

S 51 Johansson G, Sietnieks A, Janssens F, et al. Recombinant human growth hormone treatment in short children with chronic renal disease, before transplantation or with functioning renal transplants: an interim report on five European studies. *Acta Paediatr Scand Suppl.* 1990; 370: 36-42; discussion 43.

S 52 Fine RN, Koch VH, Boechat MI, at al.. Recombinant human growth hormone (rhGH) treatment of children undergoing peritoneal dialysis. *Perit Dial Int.* 1990; 10: 209-214.

S 53 Tönshoff B, Mehls O, Schauer A, Heinrich U, Blum W, Ranke M. Improvement of uremic growth failure by recombinant human growth hormone. *Kidney Int Sup*pl. 1989; 27: 201-204.

S 54 Koch VH, Lippe BM, Nelson PA, Boechat MI, Sherman BM, Fine RN. Accelerated growth after recombinant human growth hormone treatment of children with chronic renal failure. *J Pediatr*. 1989; 115: 365-371.

S 55 Lippe B, Fine RN, Koch VH, Sherman BM. Accelerated growth following treatment of children with chronic renal failure with recombinant human growth hormone (somatrem): a preliminary report. Acta Paediatr Scand Suppl. 1988; 343: 127-131.

S 56 Gil S, Aziz M, Adragna M, Monteverde M, Belgorosky A. Near-adult height in male kidney transplant recipients started on growth hormone treatment in late puberty. *Pediatr Nephrol*. 2018; 33: 175-180.

S 57 Gil S, Vaiani E, Guercio G, et al.. Effectiveness of rhGH treatment on final height of renal-transplant recipients in childhood. *Pediatr Nephrol* 2012; 27:1005–1012.

S 58 Berard E, Andre JL, Guest G, et al.. Long-term results of rhGH treatment in children with renal failure: Experience of the french society of pediatric nephrology. *Pediatr Nephrol.* 2008; 23: 2031-2038.

S 59 Nissel R, Lindberg A, Mehls O, Haffner D, Pfizer International Growth Database (KIGS) International Board. Factors predicting the near-final height in growth hormone-treated children and adolescents with chronic kidney disease. *J Clin Endocrinol Metab*. 2008; 93: 1359-1365.

S 60 Seikaly MG, Salhab N, Warady BA, Stablein D. Use of rhGH in children with chronic kidney disease: lessons from NAPRTCS. *Pediatr Nephrol.* 2007; 22:1195-1204.

S 61 Fine RN, Stablein D. Long-term use of recombinant human growth hormone in pediatric allograft recipients: a report of the NAPRTCS Transplant Registry. *Pediatr Nephrol.* 2005; 20: 404-408.

S 62 Crompton C, Australian and New Zealand Paediatric Nephrology Association. Long-term recombinant human growth hormone use in Australian children with renal disease. *Nephrology* 2004; 9:325-330.

S 63 Hokken-Koelega AC, Nauta J, Lilien M, Ploos van Amstel J, Levenko N. Long-term growth hormone treatment in children with chronic renal failure. *Pediatr. Nephrol.* 2004; 19:C38: S04.33.

S 64 Fine RN, Sullivan EK, Tejani A. The impact of recombinant human growth hormone treatment on final adult height. *Pediatr Nephrol*. 2000; 14: 679-681.

S 65 Haffner D, Schaefer F, Nissel R, Wühl E, Tönshoff B, Mehls O. Effect of growth hormone treatment on the adult height of children with chronic renal failure. German Study Group for Growth Hormone Treatment in Chronic Renal Failure. *N Engl J Med* 2000; 28; 343: 923-930.

S 66 Janssen F, Van Damme-Lombaerts R, Van Dyck M, et al.. Impact of growth hormone treatment on a belgian population of short children with renal allografts. *Pediatr Transplant*. 1997; 1: 190-196.

S 67 Callis LL, del Valle CJ, Garcia L, et al. Randomized open multicentre study to assess treatment with recombinant human growth hormone in children with low height and chronic renal insufficiency [abstract]. *An Esp Pediatr*. 1996; 45: 328.
